# A novel role for the 3′-5′ exoribonuclease Dis3L2 in controlling cell proliferation and tissue growth

**DOI:** 10.1080/15476286.2016.1232238

**Published:** 2016-09-14

**Authors:** Benjamin P. Towler, Christopher I. Jones, Kirsty L. Harper, Joseph A. Waldron, Sarah F. Newbury

**Affiliations:** Brighton and Sussex Medical School, University of Sussex, Brighton, UK

**Keywords:** *Drosophila*, RNA-seq, RNA stability, wing imaginal discs, XRN1

## Abstract

In a complex organism, cell proliferation and apoptosis need to be precisely controlled in order for tissues to develop correctly. Excessive cell proliferation can lead to diseases such as cancer. We have shown that the exoribonuclease Dis3L2 is required for the correct regulation of proliferation in a natural tissue within the model organism *Drosophila melanogaster*. Dis3L2 is a member of a highly conserved family of exoribonucleases that degrade RNA in a 3′-5′ direction. We show that knockdown of *dis3L2* in the *Drosophila* wing imaginal discs results in substantial wing overgrowth due to increased cellular proliferation rather than an increase in cell size. Imaginal discs are specified in the embryo before proliferating and differentiating to form the adult structures of the fly. Using RNA-seq we identified a small set of mRNAs that are sensitive to Dis3L2 activity. Of the mRNAs which increase in levels and are therefore potential targets of Dis3L2, we identified 2 that change at the post-transcriptional level but not at the transcriptional level, namely *CG2678* (a transcription factor) and *pyrexia* (a TRP cation channel). We also demonstrate a compensatory effect between Dis3L2 and the 5′-3′ exoribonuclease Pacman demonstrating that these 2 exoribonucleases function to regulate opposing pathways within the developing tissue. This work provides the first description of the molecular and developmental consequences of Dis3L2 inactivation in a non-human animal model. The work is directly relevant to the understanding of human overgrowth syndromes such as Perlman syndrome.

## Abbreviations

Dis3: Defective in sister chromatid rejoining 3
Dis3L2: Defective in sister chromatid rejoining 3 like 2
XRN1: Exoribonuclease-1
RNA-seq: RNA-sequencing

## Introduction

Regulation of cell proliferation is of crucial importance to all multicellular organisms. Cells must proliferate throughout development in order for the organism to grow to adult size. Proliferation must also occur to repair damaged areas during the process of wound healing. Control of proliferation is therefore vitally important to allow individual animals and their constituent organs to grow to their correct sizes, as well as to maintain symmetry between the left and right sides of an animal.^1^ In an epithelial tissue subjected to damage, cell proliferation can be stimulated by apoptosis to heal the wound, demonstrating a balance between apoptosis and proliferation.^2^ However, uncontrolled cell proliferation is a hallmark of cancer with many genes involved in growth and proliferation implicated in cancer progression.^3^

This paper describes the discovery of a new player in the control of cell proliferation within a tissue, namely the exoribonuclease Dis3L2. Dis3L2 is a 3′-5′ exoribonuclease and is a member of the highly conserved RNaseII/RNB family of 3′-5′ exoribonucleases.^4^ Prokaryotes and some eukaryotes such as *S. cerevisiae* harbour a single member of this family (Dis3) whereas other eukaryotes including *S. pombe* and *Drosophila melanogaster* encode 2 enzyme paralogues (Dis3 and Dis3L2).^5^ In humans, there is an additional paralogue, Dis3L1. Dis3L2, unlike Dis3 and Dis3L1, does not contain the N-terminal PIN domain^5,6^ which confers endoribonucleolytic activity to Dis3, and tethers it to the exosome, a multicomponent protein complex. Recent work has shown that Dis3L2 is primarily cytoplasmic and acts independently of the exosome to degrade both mRNAs and non-coding RNAs.^5-7^ In humans and *S. pombe*, this often occurs following the addition of a polyuridine (polyU) tract to the 3′ end of the transcript, which provides a binding site for Dis3L2 to initiate/reinitiate 3′-5′ decay.^5,8,9^ miRNAs such as *pre-let-7* have also been shown to be poly-uridylated and then degraded by Dis3L2 in human cells and mouse embryonic stem cells.^10,11^ In human cells, Dis3L2 has been shown to degrade *miR-27a* by target RNA-directed miRNA degradation and to associate with Ago2 in the RNA induced silencing complex (RISC).^7^

The clinical importance of Dis3L2 is demonstrated by its association with Perlman syndrome and Wilms' tumor susceptibility.^12,13^ Perlman syndrome is a congenital overgrowth condition which is inherited in an autosomal recessive manner.^14^ Affected children display foetal gigantism, abnormal enlargement of organs (e.g. kidneys), facial abnormalities, neurodevelopmental delay and high neonatal mortality. Histological examinations reveal nephroblastomatosis, which is an important precursor for Wilms' tumor. Germline mutations in these children are consistent with a loss of function of Dis3L2.^12^ In addition, a truncation of the *DIS3L2* locus has been associated with a Marfan-like syndrome with skeletal overgrowth.^15^ Dis3L2 is also likely to be important in sporadic Wilms' tumor as 30% of these tumors (6/20) show partial or complete *DIS3L2* deletion.^12^ Understanding the cellular and molecular pathways controlled by Dis3L2 in a well characterized model system such as *Drosophila melanogaster* is likely to shed light on the molecular basis of these diseases.

In this paper we show, for the first time, a role for Dis3L2 in the proliferation of imaginal disc cells during *Drosophila melanogaster* development. Imaginal discs are specified in the embryo before proliferating and differentiating during larval and pupal stages respectively to form the adult structures of the fly (e.g., wings, legs). Wing imaginal discs provide an excellent model system for the study of growth homeostasis because the cells being studied are in their natural context of a developing organism, rather than as immortalized tissue culture cells. Here we show that depletion of *dis3L2* leads to increased proliferation of cells in the wing imaginal discs, without an increase in cell size, resulting in larger wings in the adult fly. Using RNA-seq, we have evidence that Dis3L2 regulates the expression of a discrete set of transcripts including a mRNA encoding a cation channel. In addition, we show that the increase in proliferation caused by depletion of *dis3L2* can be compensated with mutations for the gene encoding the 5′-3′ exoribonuclease Pacman/Xrn1. Therefore, these 2 ribonucleases, which degrade RNA in opposite directions, appear to affect the antagonistic pathways of apoptosis and proliferation. Since Pacman and Dis3L2 are highly conserved throughout evolution, it is likely that they will have similar functions in other multicellular organisms.

## Results

### Dis3L2 is not required for *Drosophila melanogaster* viability

We have previously shown that ubiquitous knockdown of Dis3 results in complete lethality at or prior to the L2 larval stage.^16^ To test whether Dis3L2 (CG16940) is required for viability, ubiquitous GAL4 drivers were used to knockdown *dis3L2* throughout the whole organism. Two separate *UAS-dis3L2*^*RNAi*^ lines, each targeting different sequences within both isoforms of the *dis3L2* gene were used for each *GAL4* driver. Ubiquitous knockdown using *tubulin-GAL4* (*tub-GAL4*), *actin-GAL4* (*act-GAL4*) or *daughterless-GAL4* (*da-GAL4*) drivers resulted in a strong, 9.7-fold knockdown of *dis3L2* (to 10% of its level in controls). This was not associated with any lethality (Fig. S1A-B) and shows that Dis3L2, unlike Dis3, is dispensable for *Drosophila* viability. These findings are consistent with previous data from *S. pombe*^5^ and human cell lines^6,9^ which also show no effect on cell viability following *dis3L2* depletion.

### Loss of *dis3L2* in the wing imaginal discs results in wing overgrowth

**Figure 1.**
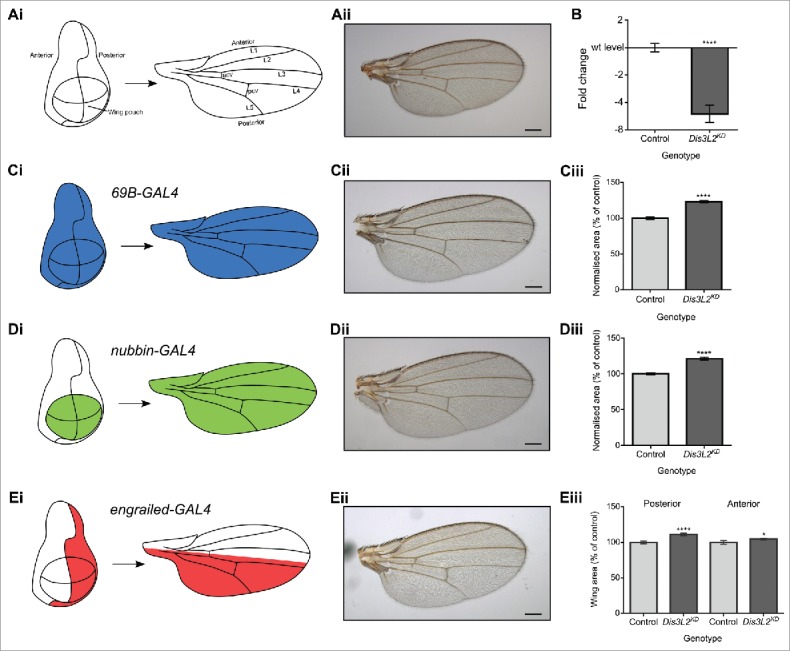
Knockdown of *dis3L2* in the wing imaginal disc results in specific overgrowth. (A) Representation of the developmental axis and regions of the *Drosophila melanogaster* wing and wing imaginal disc together with a parental control wing (*UAS-dis3L2*^*RNAi*^). (B) Knockdown of *dis3L2* throughout the wing imaginal disc using the *69B-GAL4* driver results in 5.2-fold knockdown (to 20% the level of controls) at the RNA level compared to the parental controls. n ≥ 10, error bars represent SEM, **** = p < 0.0001. (C) Knockdown of *dis3L2* throughout the wing imaginal disc using the *69B-GAL4* driver results in significant overgrowth of the wing (23%) compared to controls when wing area is normalized to fly mass. (D) Knockdown of *dis3L2* within the wing pouch of the wing imaginal disc using the *nub-GAL4* driver results in significant overgrowth (21%) of the wing compared to controls when wing area is normalized to fly mass. For (C) and (D), the “control” is the mean of the parental genotypes plus a *UAS-EGFP*^*RNAi*^ driven by the respective GAL4 driver, none of which were significantly different from each other. n ≥ 18, error bars represent 95% confidence limits, **** = p < 0.0001. (E) Restricting knockdown of *dis3L2* to the posterior region of the wing using the *en-GAL4* driver results in specific overgrowth of the posterior compartment (11%). n ≥ 20, error bars represent 95% confidence limits, **** = p < 0.0001, * = p < 0.05. Representative images of a male wing are shown in each case, scale bar = 200 µm.

We used the wing imaginal disc as a specific tissue to explore the role of Dis3L2 in tissue growth and development. The wing imaginal disc is divided into distinct regions with the wing pouch region giving rise to the wing blade (Fig. 1A) and the tip of the wing disc forming part of the thorax. Restricted knockdown was achieved using GAL4 drivers with specific expression within the wing imaginal disc (Fig. 1). Depletion of *dis3L2* throughout the wing imaginal disc (UAS-*dis3L2*^*RNAi*^*/+ ;69B-GAL4/+*) resulted in a 5.2-fold knockdown of *dis3L2* mRNA (to 20% of its level in controls)(Fig. 1B). This knockdown resulted in wings that are 123% the size of controls (Fig. 1C). However, the *69B-GAL4* driver also drives in the epidermis, together with the eye and ventral thoracic imaginal discs.^24^ To ensure the observed overgrowth was due to an effect within the wing disc a more specific *GAL4* driver was used. Use of the *nubbin-GAl4* driver (*nub-GAL4*), which drives expression within the wing pouch of the disc, to knockdown *dis3L2* also resulted in wings that were 21% larger than parental controls (Fig. 1D). To ensure the apparent overgrowth of the wings was not simply due to an increase in the size of the knockdown flies, individual flies were weighed and the wing area was normalized to the mass of the fly. Normalization of wing area made no difference to the overgrowth observed (Fig. 1C-D). For the above experiments, driving the expression of an unrelated shRNA (*UAS-EGFP*^*RNAi*^) did not result in an increase of wing size compared to controls. Use of a second *UAS-dis3L2*^*RNAi*^ line also gave identical results (Fig. S1C-D). These data therefore provide strong evidence that reduced expression of *dis3L2* within the wing imaginal discs results in the overgrowth of the adult wings.

To provide further evidence that *dis3L2* knockdown results in wing overgrowth, we used an *engrailed-GAL4* (*en-GAL4*) driver, which drives expression in the posterior compartment of the wing, so that the anterior compartment of the wing provides an internal control for wing size. Depletion of *dis3L2* using *en-GAL4* results in a significant overgrowth of the posterior region of the wing (Fig. 1E). The anterior area also showed a very slight (2.7%) but statistically significant overgrowth. However, this is most likely a knock-on effect of the 11.3% overgrowth observed in the posterior compartment. To ensure it is not only the posterior compartment that is susceptible to overgrowth we measured the anterior and posterior areas of the *nub-GAL4* driven knockdown wings and as expected the anterior and posterior areas showed comparable overgrowth of 24.9% and 18.5%, respectively (Fig. S1E). Therefore, it appears that Dis3L2 functions to control the area of the *Drosophila* wing.

### The effect of Dis3L2 on overgrowth is not consistent across the whole fly

To assess if wing disc tissue is particularly sensitive to the loss of *dis3L2* we further examined the flies with ubiquitous *dis3L2* knockdown (RNAi driven by *act-GAL4, tub-GAL4* or *da-GAL4*). Although the knockdown flies had significantly larger wings than parental controls (Fig. S2A-C), they were not significantly larger in terms of mass (Fig. S2D). This shows that the knockdown of *dis3L2* throughout the organism does not induce overgrowth of the whole fly. Therefore, it appears that there is a level of tissue specificity in the activity of Dis3L2, which would be consistent with the phenotypes of Perlman syndrome specifically affecting overgrowth of specific organs such as the liver and kidney.

### *dis3L2* depletion results in increased cell number rather than increased cell size

**Figure 2.**
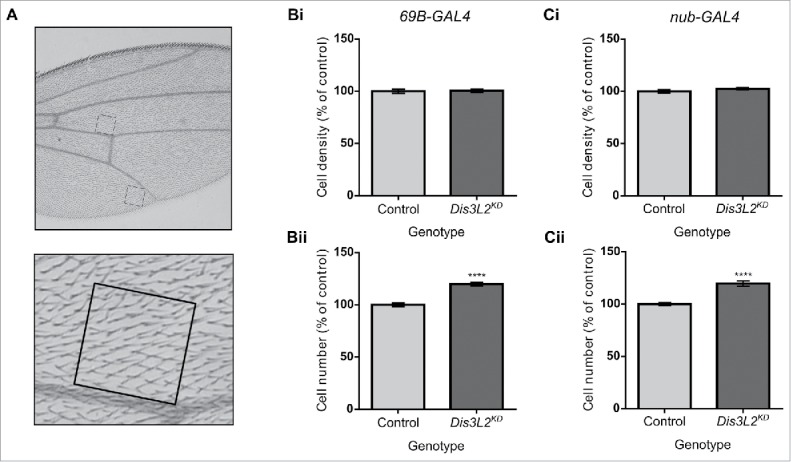
Wing overgrowth is due to an increase in cell number rather than increased cell size. (A) Representation of the 0.1mm^2^ regions of the wing used to calculate the number of cells within the wing. Blown up image shows the single hairs protruding from each cell that were counted to give the number of cells. (B-C) Knockdown of *dis3L2* throughout the wing imaginal disc using *69B-GAL4* (B) and *nub-GAL4* (C) results in an increase in cell number (ii) rather than an increase in cell size (i) compared to controls. In each case, the “control” is the average of the parental genotypes plus a *UAS-EGFP*^*RNAi*^ driven by the respective GAL4 driver, none of which were significantly different from each other. For (B) and (C) n ≥ 10, error bars represent SEM, **** = p < 0.0001.

The overgrowth phenotype observed could occur in 2 ways; either an increase in cell number (hyperplasia) or an increase in cell size (hypertrophy). To determine which of these 2 processes was occurring, we counted the total number of cells within the wing of the control and *dis3L2* knockdown flies. Since a single hair protrudes from each cell of the wing, the number of hairs can be counted to give an accurate count of the number of cells within the wing. The average number of hairs within 0.1mm^2^ regions (representing both the anterior and posterior domains) was calculated (Fig. 2A). This, along with the area of the wing was then used to estimate the total number of cells as well as their size. We observed that while the density of cells within the 0.1mm^2^ regions remained unchanged (Fig. 2Bi and Ci), indicating no change in cell size, there were 119% the number of cells in the *dis3L2-*depleted wings compared to controls (Fig. 2Bii and Cii). This indicates a hyperplasic response rather than hypertrophy of the wing. Therefore, Dis3L2 seems to play a role in controlling the proliferation of cells within the wing imaginal disc.

### The proliferative effect of *dis3L2* knockdown occurs early in development

To determine the developmental time point at which the proliferative effect of *dis3L2* knockdown is observed we used the temperature sensitive GAL80^ts^ system that allows temperature controlled knockdown of *dis3L2*.^18^ Maintaining the flies at the permissive temperature of 19°C allows GAL80^ts^ mediated repression of the *UAS-GAL4* system, therefore inhibiting any knockdown. However, moving the flies to 29°C results in inhibition of GAL80^ts^ and subsequent knockdown of *dis3L2*. Our previous results^19^ have shown the perdurance of the system to be between 24 and 32 hours. For these experiments we used the *69B-GAL4* wing disc driver and progeny cultured at 19°C and 29°C as negative and positive controls respectively.

**Figure 3.**
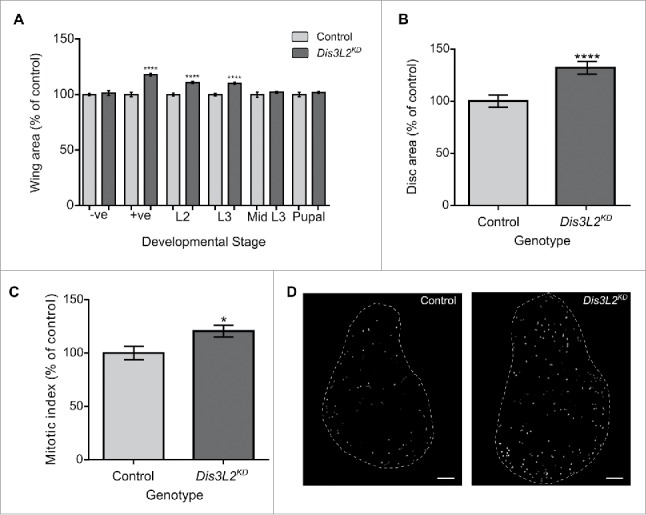
Knockdown of *dis3L2* results in increased proliferation within the wing imaginal disc leading to overgrowth of the disc. (A) Using the GAL80^ts^ system to control the developmental timings of *dis3L2* knockdown throughout the wing imaginal disc revealed that wing overgrowth is due to processes occurring during early *Drosophila* development. –ve represents flies that were maintained at the permissive 19°C temperature throughout their development. +ve represents flies that were maintained at the active 29°C temperature throughout their development. The positive control (+ve), start of L2 and start of L3 timings show a significant increase in adult wing area (**** = p < 0.0001) compared to parental controls. The negative control (−ve), mid L3 and start of pupal timings show no significant increase in wing area of knockdown flies. “Control” represents the mean wing area of the 2 parental controls. n ≥ 37, error bars represent 95% confidence limits. (B) Knockdown of *dis3L2* in the wing imaginal disc using the *69B-GAL4* driver results in significant overgrowth of the disc compared to controls. *Dis3L2*^*KD*^ late L3 wing discs are 132.0% the size of parental control discs. n ≥ 21, error bars represent 95% confidence limits, **** = p < 0.0001. (C) *Dis3L2*^*KD*^ wing discs have a significantly higher mitotic index (20.5% increase in the percentage of cells undergoing mitosis) than control discs. n ≥ 13, * = p = 0.0241, error bars represent 95% confidence limits. For (B) and (C) “Control” shows the mean measurements of the 2 parental controls. (D) Representative images of the DeadEasy MitoGlia output following staining of late L3 wing imaginal discs with anti-Phosphohistone H3. Scale bar = 50 µm.

Knockdown of *dis3L2* at the start of the 2^nd^ instar (L2) and 3^rd^ instar (L3) larval stages resulted in significant overgrowth of the wings (Fig. 3A). However, knockdown during the mid 3^rd^ instar larval stage and at the start of the pupal stage resulted in wings of wild type size (Fig. 3A). Together, these data indicate that Dis3L2 activity is required during early larval stages to control cell number. Therefore, Dis3L2 is likely to affect transcripts controlling cell proliferation of the wing imaginal discs during the early larval stages, which subsequently affects the later development of the wing.

### Depletion of *dis3L2* results in overgrowth of the wing imaginal discs

The above GAL80^ts^ experiment predicted that Dis3L2 is required during larval development to regulate cell number in the wing imaginal discs. To test this, we dissected wing imaginal discs from late L3 larvae and measured their sizes relative to parental controls. Knockdown of *dis3L2* using the *69B-GAL4* driver resulted in discs that were 132.0% the size of their respective controls (Fig. 3B) while use of the *nub-GAL4* driver increased the size of the discs to 121.6% the size of controls (Fig. S3). The difference in the level of overgrowth between the 2 drivers is most likely due to their expression domains; *nub-GAL4* drives expression within the wing pouch whereas the *69B-GAL4* expresses throughout the entire disc.

To confirm that the overgrowth was due to an increase in cell proliferation we used phosphohistone H3 (PH3) as a marker for cells undergoing mitosis. Late L3 wing discs were stained with anti-PH3, the nuclei undergoing mitosis were counted and the mitotic index was calculated (see Materials and Methods). This indicates that a significantly higher percentage of cells are undergoing mitosis in the *dis3L2* knockdown wing discs (120.5%) compared to parental control discs (Fig. 3C-D). These data therefore show that the loss of *dis3L2* results in an increased rate of cell division within the wing imaginal disc, which in turn leads in an increase in the total area of the tissue and larger adults wings.

### RNA-sequencing shows that Dis3L2 affects the expression of a small number of genes

To identify the RNAs that are misregulated in *dis3L2* knockdown imaginal discs and are likely to cause the above phenotypes, we used genome-wide RNA-sequencing (RNA-seq). We compared wing imaginal discs where *dis3L2* was knocked down to 20% of the level in parental controls, using the *69B-GAL4* driver (*dis3L2*^*RNAi*^*/+ ;69B-GAL4/+*) (Fig. 1B) with the 2 parental stocks used to generate the knockdown flies ( *;UAS-dis3L2*^*RNAi*^; and *;;69B-GAL4*) used as controls. Total RNA was prepared from 2 biological replicates of 60 dissected wing imaginal discs per genotype and then subjected to RNA-seq. RNA-seq reads were subsequently analyzed using the cufflinks pipeline (see Materials and Methods). The number of reads obtained for each sample together with the non-default parameters used during the analysis can be found in Fig. S4.

**Figure 4.**
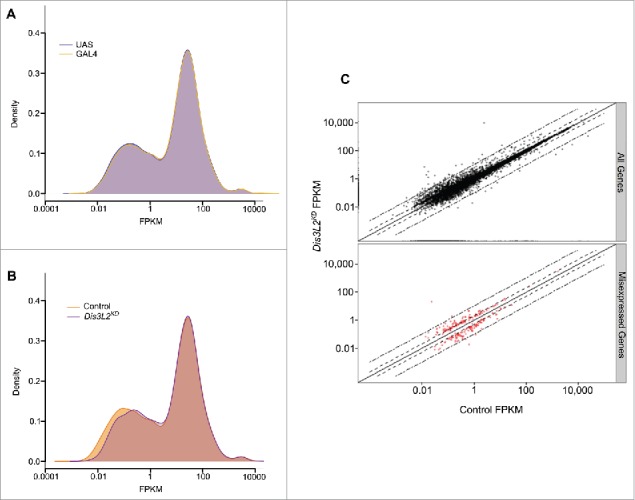
*dis3L2* knockdown largely affects transcripts expressed at low levels. (A) Kernel density plot comparing the overall gene expression profiles of the 2 parental controls (UAS = ;*UAS-dis3L2*^*RNAi*^; , GAL4 = *;;69B-GAL4*). (B) Kernel density plot comparing *dis3L2* knockdown (*Dis3L2*^*KD*^ = ;*UAS-dis3L2*^*RNAi*^*/+ ; 69B-GAL4/+*) to the grouped control (Control =;*UAS-dis3L2*^*RNAi*^; and *; 69B-GAL4*). Knockdown of *dis3L2* results in a general shift in expression of the transcripts normally expressed at low levels to a level of higher expression. The moderately and highly expressed transcripts show less variation. (C) Scatter plots showing the fold changes between control and *Dis3L2*^*KD*^ samples. All detected transcripts are shown in the top panel (black) while consistently misexpressed transcripts red are represented in the bottom panel. Dashed lines represent ± 2 fold changes whilst dot-dashed lines represent ± 10 fold changes.

We first compared the overall expression distributions for the 2 parental controls to ensure it was reasonable to group them together as a total ‘control’ group. Kernel density plots showed no significant difference (Kolmogorov-Smirnov (KS) test p = 0.555) indicating that the overall expression profiles of the 2 controls was very consistent (Fig. 4A). The four parental replicates were therefore grouped to form the “control” group. The kernel density plots show 3 major peaks comprising transcripts expressed at a very low level (far left), transcripts expressed at a moderate level (center) and a small set of transcripts expressed at a very high level (far right). Comparing the expression distributions within the control discs and the *dis3L2* knockdown discs showed a general and significant shift in expression of genes normally expressed at low levels to a level of greater expression (KS test p = 1.041e^−6^) (Fig. 4B). This is consistent with the known function of Dis3L2 and suggests that the exoribonuclease Dis3L2 normally functions to maintain a set of discrete transcripts at low levels.

Fig. 4C shows a scatter plot comparing the expression levels of individual genes in *dis3L2* knockdown discs with those of the grouped parental controls. After stringent filtering (see Materials and Methods), a total of 239 genes (2.7% of total detected) were reported as differentially expressed between the 2 groups (red dots). Therefore, Dis3L2 regulates a very small number of transcripts, which is a similar conclusion to our previous work showing that the 5′-3′ exoribonuclease Pacman has specificity for particular mRNAs.^20,21^ Consistent with the expression distributions those transcripts determined as misexpressed showed a low level of expression in parental control discs. We also observed in our sequencing data that the consistently misexpressed genes were distributed across all chromosome arms indicating there is neither a technical bias nor a chromosome bias for Dis3L2 targets (Fig. S5).

### qRT-PCR verification of the RNA-seq results shows that *pyrexia* and *CG2678* are post-transcriptionally upregulated in *dis3L2* knockdown discs

**Figure 5.**
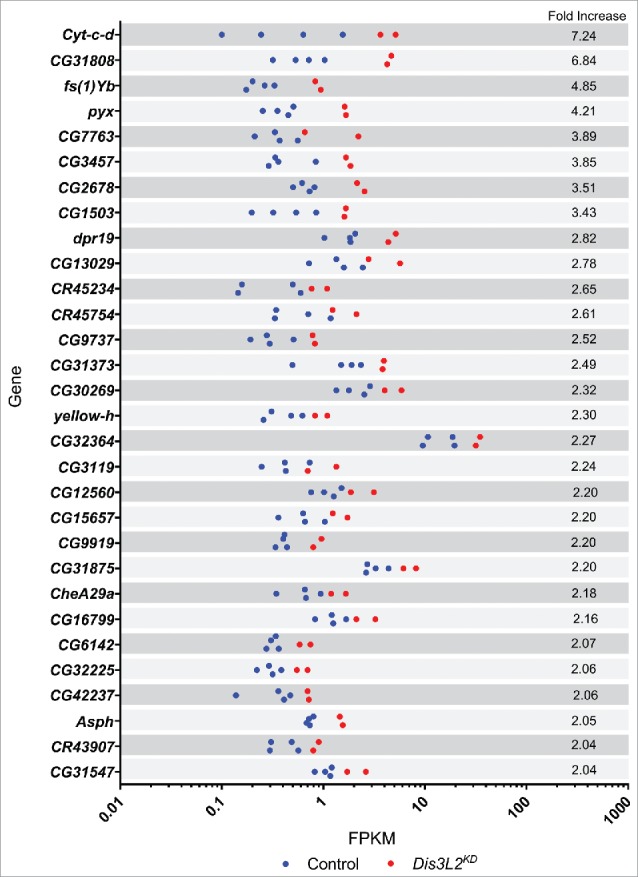
Top 30 upregulated genes. Graphical representation of the replicate consistency for the 30 transcripts showing the greatest upregulation in *dis3L2* knockdown discs with an average FPKM of more than 0.3. Knockdown replicates (*Dis3L2*^*KD*^) shown in red while control replicates are shown in blue.

Since Dis3L2 is an exoribonuclease that degrades RNAs, we would expect its candidate targets to increase in expression in the *dis3L2* knockdown wing imaginal discs to produce the observed phenotypes. A set of transcripts showed a decrease in expression in the RNA-seq data (Fig. S6). However, of the 7 validated by qRT-PCR only one showed a reproducible decrease in expression (*dis3L2*) with large biological variation suggesting these are more likely to be due to indirect effects (Fig. S7). Therefore, to validate genes sensitive to Dis3L2 activity we selected those that were the most consistently increased in expression >1.5-fold in the knockdown discs (Fig. 5). From this shortlist we selected those that showed the highest fold changes while maintaining consistency between replicates in addition to having an average FPKM of greater than 0.3. These were taken forward for validation by quantitative RT-PCR (qRT-PCR) on new biological replicates.

**Figure 6.**
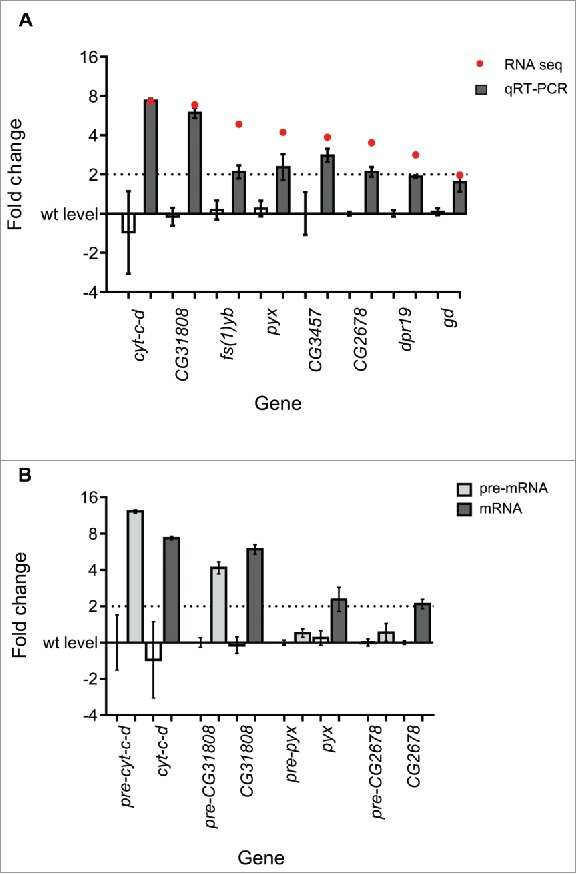
RNA-seq validation. (A) Comparison between the fold changes of the selected transcripts by RNA-seq (red dots) and qRT-PCR (gray bars). The parental control (left) and *dis3L2* knockdown (right) values are presented for each transcript. n ≥ 4, error bars represent standard error. See Table 1 for p-values. (B) *pyrexia* (*pyx)* and *CG2678* show post-transcriptional changes in gene expression as the mature mRNA (dark gray) but not the pre-mRNA (light gray) show significant increases in expression. *cyt-c-d* and *CG31808* show transcriptional changes as both the pre- (light gray) and mature (dark gray) mRNA increase in expression. The parental control (left) and *dis3L2* knockdown (right) values are presented for each transcript for both the pre- and mature mRNA. n ≥ 4, error bars represent standard error. See Table 1 for p-values.

Fig. 6A shows that the changes in expression between *dis3L2* knockdown and control discs were similar for the majority of the selected transcripts according to the 2 techniques. For example, *cytochrome-c-distal* (*cyt-c-d*) increased in expression by 7.2-fold according to RNA-seq and by 7.4-fold by qRT-PCR. Similarly, *CG31808* increased 6.8-fold and 6.0 fold by RNA-seq and qRT-PCR respectively. For other transcripts, such as *pyrexia (pyx)* and *CG2678* the change in expression detected by RT-PCR was in agreement, but more modest than by RNA-seq (Table 1). Only one gene, *CG3457*, did not show a significant change in expression by qRT-PCR. The verification of the RNA-seq by qRT-PCR demonstrates that we can have confidence in the changes presented by our RNA sequencing experiment.

**Table 1.** RNA sequencing validation summary. The p-values calculated using individual *t-tests* are presented for the fold changes identified by qRT-PCR for both the mature and precursor (pre) mRNAs for each gene. n ≥ 4.

Gene	RNA-seq FC	qRT-PCR FC	p-value (t-test)	pre-mRNA FC	p-value (t-test)
*cyt-c-d*	**7.25**	**7.36**	0.0423	**12.2**	0.0052
*CG31808*	**6.84**	**5.97**	<0.0001	**4.25**	<0.0001
*fs(1)Yb*	**4.84**	**2.11**	0.0412	**n/a**	n/a
*pyx*	**4.21**	**2.43**	0.0175	**1.2**	0.0709
*CG3457*	**3.85**	**2.85**	0.927	**n/a**	n/a
*CG2678*	**3.51**	**2.11**	<0.0001	**1.3**	0.2611
*dpr19*	**2.82**	**1.92**	<0.0001	**n/a**	n/a
*gd*	**1.97**	**1.79**	0.0072	**n/a**	n/a

To identify potential direct targets of Dis3L2 we tested whether the expression changes of 4 upregulated mRNAs (by qRT-PCR) were due to transcriptional or post-transcriptional effects. As Dis3L2 functions to degrade mRNAs at the post-transcriptional level we would expect Dis3L2 targets to be upregulated at the mRNA but not the pre-mRNA level. Those genes upregulated at the pre-mRNA level as well as the mRNA level would be expected to be indirect targets, for example due to Dis3L2 regulating an RNA encoding a transcription factor. Transcriptional changes are observed for *cyt-c-d* and *CG31808* as their pre-mRNAs increase in expression 6.6-fold and 4.3-fold respectively (compared to 7.4 and 6.0 fold increases for the mature mRNAs); indicating that the increases in expression are likely to be due to indirect effects (Fig. 6B). However, *pyrexia* and *CG2678* show a clear regulation at the post-transcriptional level as the expression of *pre-pyx* and *pre-CG2678* do not change significantly in *dis3L2* knockdown discs compared to controls, but increase in expression by 2.4 and 2.1-fold respectively at the mRNA level (Fig. 6B and Table 1). Therefore, of the 4 mRNAs tested, 2 (*pyx* and *CG2678*) are upregulated at the post-transcriptional level, suggesting that they are targets of Dis3L2.

### Dis3L2 and Pacman function to regulate opposing pathways of apoptosis and proliferation

In previous work, we showed that mutations in the gene encoding the 5′-3′ exoribonuclease *pacman* resulted in increased apoptosis.^21^ In contrast, the work outlined above shows that knockdown of the 3′-5′ exoribonuclease *dis3L2* results in increased proliferation. These results suggested to us that knockdown of *dis3L2* may rescue the effects of the *pacman* mutations. To test this hypothesis, we again utilised the *UAS-GAL4* system to knockdown *dis3L2* in the wing imaginal discs of hypomorphic (*pcm*^*5*^) and null (*pcm*^*14*^) *pacman* mutants and observed the phenotypic effects.

**Figure 7.**
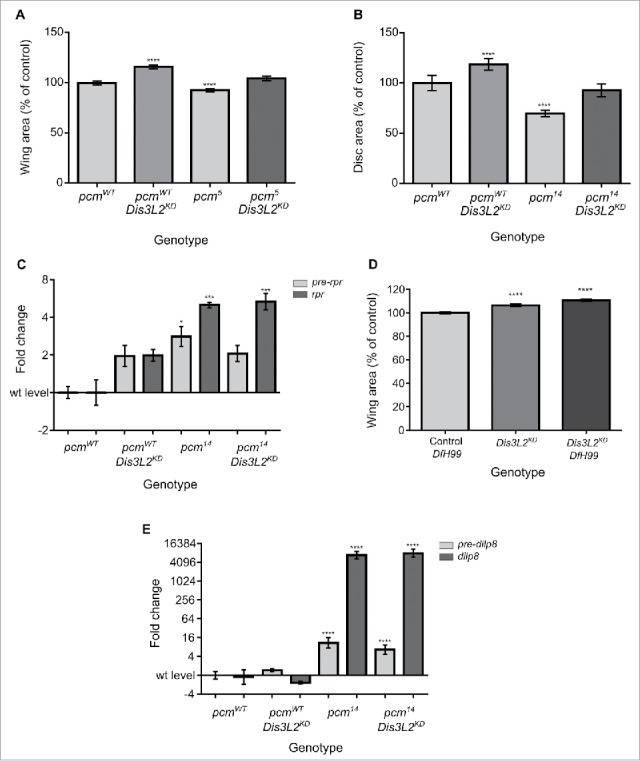
Dis3L2 and Pacman function in distinct pathways to control wing development. (A) Knockdown of *dis3L2* in the hypomorphic *pcm*^*5*^ mutant background results in a compensatory phenotype in that the wing area returns to wild type size. n ≥ 15, error bars represent 95% confidence limits, **** = p < 0.0001. (B) Knockdown of *dis3L2* in the null *pcm*^*14*^ mutant background results in a compensatory phenotype in that the wing imaginal disc area returns to wild type size. n ≥ 15, error bars represent 95% confidence limits, **** = p < 0.0001. (C) Knockdown of *dis3L2* in *pcm*^*14*^ mutant wing discs (*pcm*^*14*^ *Dis3L2*^*KD*^) has no effect on the post-transcriptional upregulation of *rpr. pre-rpr* (light gray) used to detect the level of transcription of *rpr*. n ≥ 3, error bars represent SEM, *** = p < 0.001, * = p < 0.05. (D) *dis3L2* knockdown (*Dis3L2*^*KD*^) has a larger effect on wing overgrowth than the *Df(3L)H99* mutation as a heterozygote (Control *Df(3L)H99*). However, knocking down *dis3L2* in a heterozygous *Df(3L)H99* background (*Dis3L2*^*KD*^ *Df(3L)H99)* results in an additive effect where the wings are significantly larger than *dis3L2* knockdown wings alone. n ≥ 18, error bars represent 95% confidence limits, **** = p < 0.0001. (E) Knockdown of *dis3L2* in *pcm*^*14*^ mutant wing discs (*pcm*^*14*^ *Dis3L2*^*KD*^) has no effect on the post-transcriptional upregulation of *dilp8. pre-dilp8* (light gray) used to detect the level of transcription of *dilp8*. n ≥ 3, error bars represent SEM, **** = p < 0.0001. Full details of the genotypes are provided in the Materials and Methods section.

The hypomorphic *pcm*^*5*^ mutation has been shown to have 66% of the activity of the wild-type allele resulting in a small wing phenotype.^21,22^ Knockdown of *dis3L2* throughout the wing imaginal discs in a *pcm*^*5*^ mutant background (*pcm*^*5*^*/Y ; UAS-dis3L2*^*RNAi*^*/+ ; 69B-GAL4/+*) results in wings that are not significantly different in size from control wings (Fig. 7A). This shows that the phenotypic effects of the *dis3L2* knockdown (i.e. proliferation) compensate for the phenotypic effects of the *pcm*^*5*^ mutation (i.e., apoptosis). To test this striking effect further, we also knocked down *dis3L2* throughout the wing imaginal discs in the *pacman* null mutant, *pcm*^*14*^. The *pcm*^*14*^ mutation results in small wing imaginal discs leading to a developmental delay and eventual pupal lethality.^21^ In confirmation of the above results, *dis3L2* knockdown in a *pcm*^*14*^ mutant background (*pcm*^*14*^*/Y ; UAS-dis3L2*^*RNAi*^*/+ ; 69B-GAL4/+*) results in late L3 wing imaginal discs of normal size, again showing a compensatory effect between Dis3L2 depletion and the *pcm*^*14*^ mutation (Fig. 7B).

In a previous paper,^21^ we showed that the *pcm*^*14*^ null mutation results in an increase in apoptosis due to the post-transcriptional upregulation of the pro-apoptotic genes *hid* and *reaper (rpr)*. In *Drosophila*, the key activators of the intrinsic apoptosis pathway are the Hid, Grim and Reaper proteins.^23^ To determine whether Dis3L2 derives its compensatory effects from downregulation of *rpr* expression we compared the levels of this RNA in the wing imaginal discs of *pcm*^*14*^ mutants with or without additional *dis3L2* knockdown. As shown in Fig. 7C, levels of *rpr* mRNA are very similar in both cases indicating Dis3L2 does not play a role in the regulation of *rpr* expression. Confirmation is provided by results showing that the *dis3L2* knockdown alone has no significant effect on either *rpr* pre-mRNA or mature mRNA according to qRT-PCR (Fig. 7C) or RNA-seq (1.07 fold change). Similarly, the knockdown of *dis3L2* within the wing imaginal disc has no effect on the expression of *hid* or *grim* mRNA (Fig. S8).

To confirm that knockdown of *dis3L2* does not affect apoptosis via the Hid, Grim or Reaper pathway, we made use of the *Df(3L)H99* deficiency, where these 3 genes (which are located together on the chromosome) are deleted from the genome. Heterozygous *Df(3L)H99/+* have larger wings than wild-type controls due to reduced apoptosis in the wing discs (Fig. S9). If *dis3L2* knockdown was to inhibit the Hid, Grim and Rpr apoptotic pathway we would expect knockdown of *dis3L2* in a heterozygous *Df(3L)H99* background to result in wings no different in area to the *Df(3L)H99/+* controls or when *dis3L2* is knocked down in a wild-type background. In contrast, this knockdown (; *UAS-dis3L2*^*RNAi*^*/+ ; Df(3L)H99/69B-GAL4*) results in a significant increase in wing area compared to either *Df(3L)H99/+* controls or when *dis3L2* is knocked down in a wild-type background (*UAS-dis3L2*^*RNAi*^*/+ ; 69B-GAL4/+*) (Fig. 7D). Therefore, Dis3L2 acts independently of the Hid, Grim and Reaper induced Caspase pathway and controls proliferation rather than preventing apoptosis. The lack of redundancy between these exoribonucleases is further illustrated by the fact that following the loss of one decay enzyme (Dis3L2) the cell does not increase the transcription of mRNAs encoding either of the other major RNA decay enzymes, Pacman and Dis3 to compensate (Fig. S10).

Although depletion of *dis3L2* rescues wing disc area in *pcm*^*14*^ mutants, the *dis3L2*^*KD*^, *pcm*^*14*^ mutant larvae are still developmentally delayed. We have previously shown that the 5′-3′ exoribonuclease Pacman post-transcriptionally regulates the expression of the insulin-like peptide Dilp8 resulting in a developmental delay.^20^ To determine whether *dilp8* levels are also regulated by Dis3L2, we used qRT-PCR to assess the levels of *dilp8* pre-mRNA and mature mRNA after *dis3L2* knockdown with or without the *pcm*^*14*^ mutant background. Fig. 7E shows that *dilp8* mRNA levels increase approximately 8000 fold in the *dis3L2* knockdown *pcm*^*14*^ mutant discs (*pcm*^*14*^*/Y ; UAS-dis3L2*^*RNAi*^*/+ ; 69B-GAL4/+*) which is not significantly different to the increase in *pcm*^*14*^ discs (*pcm*^*14*^*/Y ; UAS-dis3L2*^*RNAi*^ *;)*. These data further emphasizes the specificity of transcripts being targeted to particular exoribonucleases. Taken together these data indicate that Dis3L2 and Pacman have opposing functions of proliferation and apoptosis within cells, which are achieved through the degradation of different and specific RNA targets.

## Discussion

In this study we show that the 3′-5′ exoribonuclease Dis3L2 controls organ size in *Drosophila melanogaster*. Knockdown of *dis3L2* in the *Drosophila* wing imaginal disc results in substantial wing overgrowth due to increased cellular proliferation during the larval stages. Using RNA-seq we have identified a small set of mRNAs that are sensitive to Dis3L2 activity, with only 239 genes changing in expression >1.5-fold out of the 8837 detected. Of the mRNAs that increase in expression upon *dis3L2* knockdown, and are therefore likely to be targets of Dis3L2, we identified 2 that change at the post-transcriptional level but not at the transcriptional level, namely *CG2678* and *pyrexia*. We also demonstrate a compensatory effect between Dis3L2 and the 5′-3′exoribonuclease Pacman where knockdown of *dis3L2* in a *pacman* mutant background results in a normal wing size. Loss of Pacman results in increased apoptosis^21^ while *dis3L2*-depletion promotes proliferation demonstrating that these 2 exoribonucleases function to regulate opposing pathways within the developing tissue.

The results described in this paper are in agreement with previous reports that Dis3L2, unlike its paralogue Dis3, is dispensable for organism viability,^5,6,9^ indicating the specific roles of these highly related exoribonucleases. However, we cannot at present rule out that Dis3L2 is required during very early developmental stages, as ubiquitous knockdown with the GAL4 drivers we have used is not effective prior to the syncytial blastoderm stage when the majority of zygotic transcription commences. Our data are also in line with a previous paper showing that Dis3L2 depletion in HeLa cells results in increased cell proliferation.^12^ However, we do not have any evidence that Dis3L2 knockdown inhibits apoptosis, as has previously been reported in HeLa cells.^9^ Since our study uses normal cells in the context of a natural tissue rather than as immortalised cells, it is not surprising that our results differ from the above study. The use of a developing organism has also allowed us to show that Dis3L2 normally represses cell proliferation during the early developmental stages of wing disc development (in L2 and early L3 larvae) as depletion in later stages (mid L3 and early pupal) has no effect on proliferation, even though Dis3L2 is normally expressed throughout wing disc development.^24^ In addition, in common with the effect of Dis3L2 in human diseases such as Perlman syndrome, our data show that Dis3L2 has specific effects on certain tissues as ubiquitous knockdown affects wing size but does not increase the mass of the whole fly. It is possible that Dis3L2 predominantly affects tissues that are regenerative and/or particularly proliferative such as the kidneys and liver in humans and wing imaginal discs in flies.

The use of RNA-seq as an unbiased global approach allowed us to characterize the ways that Dis3L2 functions to control gene expression. Three main groups of transcripts in L3 wing imaginal discs were identified: those that are expressed at very high levels, those at moderate levels and the Dis3L2 sensitive genes at a very low level. This pattern of gene expression is consistent with our previous work.^20^ The general low level of expression in control wing imaginal discs for the Dis3L2 sensitive genes suggests a role for Dis3L2 in maintaining the expression of these specific genes at low levels, presumably to prevent the overgrowth of the developing wing imaginal discs and wings. Our results show that the depletion of *dis3L2* results in the deregulation of a small number of transcripts. The small number may be due to silencing efficiency, but is more likely to represent the substrates most sensitive to *dis3l2* depletion. We have shown that the upregulation of the transcripts that increase in expression occurs either at transcriptional level or at the post-transcriptional level. The increase in levels of *cyt-c-d* and *CG31808* at the transcriptional level suggest that depletion of *dis3L2* results in an increase in activity of one or more transcriptional activators, which in turn enhance expression of these transcripts. Interestingly, *cyt-c-d and CG31808* neighbor each other on the left arm of chromosome 2 with their 5′UTRs overlapping. It is therefore possible that their increased expression is due to transcriptional co-regulation. Chromatin immunoprecipitation experiments from the modENCODE consortium^25^ have identified binding sites for transcription factors such as Trithorax-like (Trl), Dichaete (D), Distal-less (Dll) and Dorsal (dl) within the overlapping region of *cyt-c-d* and *CG31808*. Although the expression of these transcription factors at the RNA level remains unchanged following *dis3L2* knockdown (Fig. S11) it is possible that their protein activity is altered following the loss of Dis3L2 in the discs, which in turn drives the observed increase in expression of *cyt-c-d* and *CG31808*. Expression of *cyt-c-d* and *CG31808* do not change significantly in *pacman* mutants,^20^ again demonstrating the specificity of Dis3L2 on these cellular pathways.

The finding that *cyt-c-d* is upregulated in *dis3L2* knockdown tissue is interesting and suggests a mechanism whereby *dis3L2* controls proliferation. Cytochrome C (Cyt c) is an essential component of the mitochondrial electron transport chain, which is involved in the generation of ATP. The role of *cyt-c-d* in the generation of ATP in *Drosophila* cells is confirmed by experiments where knockdown of *cyt-c-d* using RNAi leads to reduced ATP levels.^26^ In mammalian cells, it has been shown that, under certain cellular conditions, Cyt c can be released from mitochondria and bind to the apoptosome complex for activation of initiator caspase-9.^27^ Although *cyt-c-d* mutations (e.g. the point mutation *cyt-c-d*^*Z2-1091*^) result in inhibition of apoptosis during development of the retina in *Drosophila*,^28,29^ the mechanism whereby this may occur in *Drosophila* is controversial.^27^ Since our experiments show no effect of *dis3l2* knockdown on apoptosis, a simple hypothesis is that increased *cyt-c-d* levels result in an increase of ATP in the cell. Since ATP is required for the generation of ribosomes, and ribosomes are required for cell proliferation, the increase in *cyt-c-d* is consistent with an increase in cell proliferation.

Our experiments have identified potential Dis3L2 targets, *CG2678* and *pyx*, which increase post-transcriptionally upon *dis3L2* depletion. These changes in expression have been shown to be specific to the loss of Dis3L2 as they are not seen in *pcm* mutant wing imaginal discs.^20^ *CG2678* is predicted to encode a transcription factor with homology to the human Krüppel C2H2-type zinc finger proteins ZNF37A and ZNF768.^24^ The biological functions of these proteins are not known. *pyrexia* (*pyx; dTRPA2; CG17142*) encodes a transient receptor potential (TRP) channel which mediates temperature response in *Drosophila*.^30^ The TRP family of receptors are evolutionarily conserved throughout metazoans and comprise ankyrin repeats plus a cation transport membrane domain, which is particularly permeable to potassium ions.^31^ Although the relationship between *pyx* and proliferation has not been studied, it is known that potassium ions are involved in the cell cycle and proliferation^32^ and that mutations in the gene encoding the TRP channel TRPP2 (PKD2) can result in polycystic kidney disease.^33^ Interestingly, the *Drosophila* retinoblastoma protein RBF1 and the transcription factor E2F, both of which play important roles in cell division and cell cycle regulation, bind to the promoter of *pyx* thus linking *pyx* to cell proliferation.^34^ Regulation of the potassium channel protein Pyx by Dis3L2 may represent a novel way of controlling tissue proliferation.

The findings that the 3′-5′ exoribonuclease Dis3L2 regulates proliferation while the 5′-3′ exoribonuclease Pacman controls apoptosis are novel and intriguing. Our data strongly suggest that Dis3L2 and Pacman regulate different and opposing pathways in proliferation and apoptosis, rather than both of them affecting the same pathway in opposite directions. This is demonstrated in our experiments showing that Dis3L2 is not functioning to prevent overgrowth through the regulation of the expression of the proapoptotic genes *hid, grim* and *rpr*. In contrast, our previous results^21^ showed that Pacman post-transcriptionally regulates these RNAs to inhibit apoptosis. Therefore, control of apoptosis and proliferation by these 2 exoribonucleases is achieved by regulation of independent cellular pathways. Although Pacman and Dis3L2 can control the balance of apoptosis/proliferation to determine the final size of the wing, they do not control compensatory pathways to control developmental delay. Knockdown of Dis3L2 does not affect the expression of *dilp8* or developmental delay in wild-type or *pcm* mutant backgrounds further emphasizing the distinct target specificity of these 2 exoribonucleases. This result would appear to conflict with previous experiments on human tissue culture cells, which have identified an RNA-dependent interaction between human Dis3L2 and XRN1, suggesting they target the same RNAs for degradation.^6^ A possible explanation is that many RNAs can be degraded by both the 5′-3′ and 3′-5′ pathways; however, there are a small number of transcripts which are specifically targeted by Dis3L2 or Pacman.

How is this specificity achieved? Recent research has demonstrated that Dis3L2 preferentially targets RNAs and pre-miRNAs that are tailed by the addition of uridines.^5,9-11^ This tailing is performed by uridyltranferases (TUTases) which in *Drosophila* include the uridyl transferase Tailor. Tailor is known to uridylate intron derived pre-miRNAs showing preference for substrates with a 3′ terminal Guanine.^35^ Tailor may also play a role in the uridylation of mRNA substrates and therefore may be responsible for targeting specific mRNAs for Dis3L2 degradation. A further possibility is that Dis3L2 targets could have specific instability elements within their 3′ UTRs, similar to AU rich elements used by the exosome to target mRNAs for decay.^36,37^ Finally, specificity may be conferred by binding of specific miRNAs which then induce RNA degradation. In summary, our work has led to the discovery of the importance of the 3′-5′ exoribonuclease Dis3L2 in cell proliferation plus tissue growth and shows that it controls a specific set of RNAs independent of the 5′-3′ degradation pathway. This work also demonstrates that *Drosophila* provides an excellent model system to understand the molecular basis of human overgrowth syndromes such as Perlman syndrome and Wilms' tumor.

## Materials and methods

### Fly husbandry

Fly stocks were cultivated on standard media in uncrowded conditions at 25°C unless otherwise stated. All stocks used were obtained from the Bloomington stock center unless stated otherwise.

GAL4 drivers used were: *tub-GAL4* (*P{w*^*+mC*^* = tubP-GAL4}LL7* originally from stock 5138, *y*^*1*^ w^*^ *;; P{w*^*+mC*^* = tubP-GAL4}LL7/TM6b,GFP*), *act5C-GAL4* (stock 4414, *y*^*1*^ w^*^; P{w^*+mC*^* = Act5C-GAL4}25FO1/CyO, y*^*+*^ ; ), *da-GAL4* (stock 108252, *w*^*1118*^*, P{w*^*mW.hs*^*Gal4-da.G32}UH1*), *nub-GAL4* (stock 25754; *P{UAS-Dcr-2.D}1, w*^*1118*^*; P{GawB}nub-AC-62*), *69B-GAL4* (stock 1774; w*;; P{GawB}69B) and *en-GAL4 (*kind donation from Paul Martin*, ; en-GAL4, UAS-GFPactin/CyO*;). The GAL80^ts^ stock (w*; P[tubP-GAL80^ts^]20/CyO-GFP; P{GawB}69B/TM6) was made from Bloomington stocks 7019 (w*; P[tubP-GAL80^ts^]20 ; TM2/TM6) and 1774 (w*;; P{GawB}69B).

RNAi stocks were obtained from the Vienna Drosophila RNAi Center. Stocks used were: *UAS-dis3L2*^*RNAi*^(stock v51854, *w*^*1118*^ *;P{GD9240}v51854*; and stock v100322, *;P{KK105902}VIE-260B*;). *UAS-EGFP*^*RNAi*^ was also obtained from the Bloomington stock center (stock 41557, *; P{VALIUM22-EGFP.shRNA.1}attP40*;) and used as a control. Phenotypes produced by both *UAS-dis3L2*^*RNAi*^ lines were indistinguishable from each other when driven by all drivers; all results shown are using *UAS-dis3L2*^*RNAi*^ stock v51854, except for Figs. S1 C/D and S2 Di/ii.

### Genetic crosses and genotypes

To knockdown *dis3L2* throughout the wing imaginal disc ;;*69B-GAL4* females were crossed to *;UAS-dis3L2*^*RNAi*^; males producing knockdown offspring with the genotype *;UAS-dis3L2*^*RNAi*^*/+ ; 69B-GAL4/+*. In addition to the parental controls *;UAS-EGFP*^*RNAi*^; males were also crossed to *;;69B-GAL4* females to produce control offspring *;UAS-EGFP*^*RNAi*^*/+ ; 69B-GAL4/+* which accounted for the expression of a hairpin RNAi construct.

To knockdown *dis3L2* within the wing pouch of the wing imaginal disc ;*nub-GAL4*; females were crossed to *;UAS-dis3L2*^*RNAi*^; males producing knockdown offspring with the genotype *;UAS-dis3L2*^*RNAi*^*/nub-GAL4;*. In addition to the parental controls *;UAS-EGFP*^*RNAi*^; males were also crossed to *;nub-GAL4*; females to produce control offspring (*;UAS-EGFP*^*RNAi*^*nub-GAL4*;) which accounted for the expression of a hairpin RNAi construct.

*dis3L2* knockdown was achieved in the posterior compartment of the wing imaginal disc by crossing *;en-GAL4/CyO*; females to *;UAS-dis3L2*^*RNAi*^; males to produce *;en-GAL4/UAS-dis3L2*^*RNAi*^; offspring. Due to the *en-GAL4* parents containing the CyO marker making them unsuitable for measurements the *;UAS-dis3L2*^*RNAi*^; parents were used as controls.

For the experiments presented in Fig. 7, *dis3L2* was knocked down in *pcm* mutant backgrounds. In the hypomorphic experiments (*pcm*^*5*^) the ;*UAS-dis3L2^RNAi^; and ;;69B-GAL4* parents served as controls (*pcm*^*WT*^). The *pcm*^*5*^ flies carried the genotype *pcm*^*5*^ *;UAS-dis3L2*^*RNAi*^; to account for any difference the *UAS-dis3L2*^*RNAi*^ insertion has on wing development. *pcm*^*5*^*Dis3L2*^*KD*^(*pcm*^*5*^ *;UAS-dis3L2*^*RNAi*^*/+; 69B-GAL4/+*) wings were compared to *pcm*^*5*^, *pcm*^*WT*^ and *Dis3L2*^*KD*^ *(;UAS-dis3L2*^*RNAi*^*/+ ; 69B-GAL4/+*) alone wings. We used the same approach with the null mutant (*pcm*^*14*^) experiments. *pcm*^*WT*^ larvae contained the isogenic control for *pcm*^*14*^ (50E) and the *UAS-dis3L2*^*RNAi*^ insertion (*50E ; UAS-dis3L2*^*RNAi*^;). Similarly, the *pcm*^*14*^ larvae also carried the *UAS-dis3L2*^*RNAi*^ insertion (*pcm*^*14*^*/Y ; UAS-dis3L2*^*RNAi*^;). *dis3L2* was knocked down in a *pcm*^*14*^ mutant background as follows (*pcm*^*14*^*/Y; UAS-dis3L2*^*RNAi*^*/+ ; 69B-GAL4/+*). *dis3L2* knockdown was also performed in the control, 50E background where *50E; UAS-dis3L2*^*RNAi*^*/+ ; 69B-GAL4/+* wings were assessed. There was no significant difference between *50E; UAS-dis3L2*^*RNAi*^*/+ ; 69B-GAL4/+ and ; UAS-dis3L2*^*RNAi*^*/+ ; 69B-GAL4/+* wings.

### GAL80^ts^ experiment

*; tub-GAL80*^*ts*^*; 69B-GAL4* homozygous females were crossed to *; UAS-dis3L2*^*RNAi*^; males and left to mate for 2 d at the appropriate temperature in addition to parental controls for all time points (19°C for all developmental timings except the positive control at 29°C). Flies were tipped into new vials for 8 hour egg lays. Vials for both the parental controls and the *; UAS-dis3L2*^*RNAi*^*/GAL80*^*ts*^ *; 69B-GAL4/+* flies were moved from 19°C to 29°C at the required times for each developmental stage taking into account a 24-32hr perdurance. Each comparison between knockdown and controls was made specifically between those for each time point.

### Wing mounting and measurement

Flies were aged to between 1 and 2 days old for all measurement experiments. A single wing (the ‘left-hand’ wing) was cut from each fly using microscissors and stored in isopropanol for 1 hour before being mounted in DPX (Sigma cat. no. 06522). For all experiments the controls represent the mean wing area of the parental controls together with an *EGFP*^*RNAi*^ driven by the same GAL4 drivers. All results shown were obtained with male wings, however female wings also showed the same phenotypes. Mounted wings were measured using Axiovision 4.7 on an Axioplan microscope (Carl Zeiss). *UAS-EGFP*^*RNAi*^ was driven by both *69B-GAL4* and *nub-GAL4* and when normalized to fly mass they were not significantly larger the parental controls.

### Disc dissection, mounting and measurement

Wing imaginal discs were dissected from late L3 larvae in PBS. Late L3 larvae were staged by the addition of bromophenol blue to the food (0.05%) followed by selection of larvae that had cleared the dyed food from their gut. Dissected discs were mounted on Poly-L-Lysine slides and mounted in 85% glycerol. Disc areas were measured using Axiovision 4.7 on an Axioplan microscope (Carl Zeiss).

### Immunocytochemistry

Immunocytochemistry was performed essentially as described in^38^ on late L3 wing imaginal discs. Images were taken with a Leica SP8 confocal microscope. Anti-Phosphohistone H3 (Cell Signaling, cat no. 9701) was used at 1:300 dilution. Cy3-conjugated monoclonal Donkey anti-rabbit IgG secondary antibody was used at 1:400 dilution (Jackson ImmunoResearch, cat. no.715-165-151).

### Calculation of the mitotic index

The number of nuclei undergoing mitosis were counted using the ImageJ plugin DeadEasy MitoGlia.^39^ All discs were stained with anti-Phosphohistone H3 as above under the same conditions. The mitotic index was then calculated for each disc by dividing the number of cells in M phase by the area of the disc.

### RNA extraction and qRT-PCR

RNA extractions were performed using a miRNeasy RNA extraction kit (Qiagen, cat. no. 217084), with on-column DNAse digestion (Qiagen, cat. no. 79254). RNA concentrations were measured on a NanoDrop1000 spectrophotometer (Thermo Scientific). When examining wing imaginal discs at least 30 imaginal discs were collected for each biological replicate.

For qRT-PCR, 500ng of total RNA was converted to cDNA in duplicate using a High Capacity cDNA Reverse Transcription Kit (Life Technologies, cat. no. 4368814) with random primers or oligo(dT) primers. A control “no RT” reaction (without enzyme) was performed in parallel to confirm that all genomic DNA had been degraded. qRT-PCR was performed on each cDNA replicate in duplicate (i.e. Four technical replicates), using TaqMan Universal PCR Master Mix, No AmpErase UNG (Life Technologies, cat. no. 4324018) and an appropriate TaqMan assay (Life Technologies). For custom pre-mRNA assays, the pre-mRNA sequence of the desired target area was submitted to Life Technologies' web-based Custom TaqMan Assay Design Tool as in^22^ (Fig. S12). *RpL32* (*Rp49*) gene was used for normalization.

### RNA-seq analysis and selection of consistently changed genes

RNA extraction was performed as above from 60 wandering L3 wing imaginal discs. RNA integrity was checked using an Agilent 2100 Bioanalyzer. 3µg of total RNA per replicate was sent to Oxford Gene Technology for sample preparation and sequencing. Libraries were poly(A) selected and then were run on a single HiSeq2000 lane using TruSeq v3 chemistry (Illumina) generating between 12 and 15 million paired end reads of 100bp per sample. The RNA-seq data has been deposited in the ArrayExpress repository under the accession number E-MTAB-4545.

Sequencing adaptors were identified using FastQC c0.11.2 (http://www.bioinformatics.babraham.ac.uk/projects/fastqc/) and removed using Scythe v0.993b (https://github.com/vsbuffalo/scythe). Further quality control and read trimming was achieved using Sickle v1.29 (https://github.com/najoshi/sickle). The remaining high quality reads were mapped to the *Drosophila melanogaster* genome from FlyBase r6.01^40^ using TopHat v2.0.12.^41^ Differential expression was completed and normalized FKPM values were generated using the Cufflinks pipeline.^42^ Alignment results and non-default parameters used during the analysis are shown in Fig. S4.

Due to the knockdown replicates being in duplicate the statistical output from Cufflinks could not be used. Therefore stringent filtering was applied. Only genes showing a fold change of ≥1.5-fold when compared between each knockdown and parental control replicate were selected as misexpressed genes. The Cuffdiff output for the misexpressed genes can be found in Supplemental File 1. Only those with an average fragments per kilobase per million (FPKM) of 0.3 where considered for validation by qRT-PCR due to the difficulty of reliably detecting very lowly expressed genes by qRT-PCR. The FPKM cut-off of 0.3 was chosen because this threshold has been used previously^43,44^ and it provided results which are reliable enough to be verified by qRT-PCR. Those showing the highest reliable fold changes together with a suitable average FPKM were selected for verification.

### Statistical tests

All statistical analyses were performed in either R v3.1.2 or GraphPad Prism 6. Two-sided 2-sample t-tests were used to compare the means of single test groups to single control groups. If multiple comparisons were required, a one-way ANOVA was performed with a post-test to compare the means of each possible pair of samples.

## Supplementary Material

Supplementary_Data.zip
