# Supplementary material for: A novel role for the 3′-5′ exoribonuclease Dis3L2 in controlling cell proliferation and tissue growth
Source: RNA Biol. 2016 Sep 14;13(12):1286–99. doi: 10.1080/15476286.2016.1232238 (PMC5207379; doi:10.1080/15476286.2016.1232238)
Supplement: Supplementary_Data.zip [file krnb-13-12-1232238-s001.zip › 1. Supplemental Materials Legend.docx]

**Supplemental Figure S1: Additional ubiquitous knockdown and wing measurement data.** **(A)** Knockdown of *dis3L2* driven throughout the organism using *tubulin-Gal4* (*tub-GAL4*) does not have any effect on organism survival when compared to siblings. Control genotype = ;*UAS-dis3L2^RNAi^/+ ; TM6/+, Dis3L2^KD^* genotype = ;*UAS-dis3L2^RNAi^/+ ; tub-GAL4/+* . n=276, error bars represent 95% confidence limits, ns = p>0.05. **(B)** Ubiquitous knockdown of *dis3L2* using *tub-GAL4* results in a 9.7 fold knockdown (to 10% of that of control) in whole 3^rd^ instar larvae. Control genotypes = ;*UAS-dis3L2^RNAi^;* and *;;tub-GAL4/TM6. Dis3L2^KD^* genotype = ;*UAS-dis3L2^RNAi^/+ ; tub-GAL4/+* . n≥4, error bars represent SEM, **** = p<0.0001. **(C)** Knockdown of *dis3L2* throughout the wing imaginal disc using the *69B-GAL4* driver and an alternative *UAS-dis3L2^RNAi^* (v100322) results in significant overgrowth of the wing (26%) when wing area is normalised to fly mass. Control genotypes = ;; *UAS-dis3L2^RNAi^, ;UAS-EGFP^RNAi^/nub-GAL4;* and *;nub-GAL4;*. *Dis3L2^KD^* genotype = *;nub-GAL4/+ ; UAS-dis3L2^RNAi^/+*). n≥54, error bars represent 95% confidence limits, **** = p<0.0001. **(D)** Knockdown of *dis3L2* within the wing pouch of the wing imaginal disc using the *nub-GAL*4 driver and an alternative *UAS-dis3L2^RNAi^* (v100322) results in significant overgrowth (25%) of the wing when wing area is normalised to fly mass. Control genotypes = ;; *UAS-dis3L2^RNAi^, UAS-EGFP^RNAi^*/*+ ;* *69B-GAL4/+* and *;; 69B-GAL4*. *Dis3L2^KD^* genotype = ;*; UAS-dis3L2^RNAi^/69B-GAL4*). n≥27, error bars represent 95% confidence limits, **** = p<0.0001. **(E)** Both the posterior and anterior regions of the wing are susceptible to *dis3L2* knockdown induced overgrowth when knockdown is driven by *nub-GAL4***.** Control genotypes = ;*UAS-dis3L2^RNAi^ ;* and *;nub-GAL4;. Dis3L2^KD^* genotype = ;*UAS-dis3L2^RNAi^/nub-GAL4 ;.*  n≥18, error bars represent 95% confidence limits, **** = p<0.0001.

**Supplemental Figure S2: Ubiquitous knockdown of *dis3L2* results in wing overgrowth but no change in fly mass.** Knockdown of *dis3L2* driven by *act-GAL4* **(A)**, *tub-GAL4* **(B)** or *da-GAL4* **(C)** results in significant overgrowth of the wing when normalised to fly mass. **(A)** Control = *; UAS-dis3L2^RNAi^ ;* , *Dis3L2^KD^* = ; *act-GAL4/UAS-dis3L2^RNA^ ;,* n≥12, error bars represent 95% confidence intervals, ****-p<0.0001. **(B)** Control = *; UAS-dis3L2^RNAi^ ;* , *Dis3L2^KD^* = ; *UAS-dis3L2^RNAi^/+ ; tub-GAL4/+,* n≥20, error bars represent 95% confidence intervals, **** = p<0.0001. **(C)** Control = *; UAS-dis3L2^RNAi^ ;* and ;; *da-GAL4* , *Dis3L2^KD^* = ; *UAS-dis3L2^RNAi^/+ ; da-GAL4/+,* n≥18, error bars represent 95% confidence intervals, **** = p<0.0001. **(D)**  Ubiquitous knockdown of *dis3L2* using *act-GAL4* and each of the *UAS-dis3L2^RNAi^* line (v51854) **(Di)** and (v100322) **(Dii)** does not result in an increase in fly mass. The *act-GAL4* driver was used due to the cross producing suitable sibling controls for analysis. n≥16, error bars represent 95% confidence intervals, ns = p>0.05.

**Supplemental Figure S3: Knockdown of dis3L2 in the wing pouch of the wing disc also results in overgrowth of the disc.** Knockdown of *dis3L2* driven by the *nub-GAL4* driver results in late L3 wing wing discs that are 121.6% the size of control discs. Control genotype = ; *UAS-dis3L2^RNAi^ ;* and *; nub-GAL4 ;. Dis3L2^KD^* genotype = ; *UAS-dis3L2^RNAi^/nub-GAL4 ;*. n≥21, error bars represent 95% confidence limits, **** = p<0.0001.

# Supplemental Figure S4: Additional RNA-seq information.

**Supplemental Figure S5: Consistently misregulated genes are situated across all the chromosome arms.**

**Supplemental Figure S6: Top 30 downregulated genes.** Graphical representation of the replicate consistency for the 30 transcripts showing the greatest downregulation in *dis3L2* knockdown discs with an average FPKM of more than 0.3. Knockdown replicates (*Dis3L2^KD^*) shown in red whilst control replicates are shown in blue.

**Supplemental Figure S7: Validation of 7 downregulated transcripts shows limited reproducibility**. Only *dis3L2* shows a reproducible downregulation between the RNA-seq data (red dot) and qRT-PCR (grey bar). The remaining 6 transcripts show high variation between biological replicates and therefore is likely to represent transcripts that show a large degree of natural variation. n≥4, error bars represent SEM. p>0.05 for all excluding *dis3L2* (p<0.0001).

**Supplemental Figure S8: Knockdown of *dis3L2* does not affect the expression of the pro-apoptotic genes *hid*, *grim* and *reaper*. (A)** Comparison between the fold changes of the selected transcripts by RNA-seq (red dots) and qRT-PCR (grey bars). The parental control (left) and *dis3L2* knockdown (right) values are presented for each transcript. n≥4, error bars represent standard error, p>0.05 for all.

**Supplemental Figure S9: The *Df(3L)H99* heterozygous deletion alone results in wing overgrowth.** Wing area of controls (;*UAS-dis3L2^RNAi^;*) are significantly smaller than the same control wings carrying the *Df(3L)H99* deletion ( *; UAS-dis3L2^RNAi^ ; Df(3L)H99/+*). n≥30, error bars represent 95% confidence limits, **** = p<0.0001.

**Supplemental Figure S10: There is no compensatory increase in transcription of the other major RNA decay enzymes.** **(A/B)** There is no compensatory increase in either *dis3* **(A)** or *pacman* **(B)** across the whole larvae when *dis3L2* is knocked down ubiquitously using *tub-GAL4*. Control genotypes = *;UAS-dis3L2^RNAi^;* and *;;tub-GAL4/TM6*), *Dis3L2^KD^* genotype = *;UAS-dis3L2^RNAi^/+ ; tub-GAL4/+*. **(C/D)** There is no compensatory increase in either *dis3* (**C**) or *pacman* (**D**) in the wing imaginal discs when *dis3L2* is knocked down throughout the disc using *69B-GAL4*. Control genotypes = *;UAS-dis3L2^RNAi^;* and *;;69B-GAL4*), *Dis3L2^KD^* genotype = *;UAS-dis3L2^RNAi^/+ ; 69B-GAL4/+*. For **(A)**-**(D)** n≥3, error bars represent SEM, p>0.05.

**Supplemental Figure S11:** **Transcription factors predicted to bind *cyt-c-d* and *CG31808* do not show expression changes in *dis3L2^KD^* wing imaginal discs.** There are no differences in normalised expression (FPKM) of *Dichaete (D)*, *dorsal (dl)* or *distal-less (dll)* in *dis3L2^KD^* wing imaginal discs (red dots, genotype = *; UAS-dis3L2^RNAi^/+ ; 69B-GAL4/+*) compared to control discs (blue dots, genotypes = *; UAS-dis3L2^RNAi^ ;* and  *;; 69B-GAL4*).

**Supplemental Figure S12: Custom TaqMan Assays.** pre-mRNA assays were designed by submitting 100bp of sequence 50bp either side of an intron-exon boundary. Forward and reverse primers (arrows) and probe (red line) locations and sequences are shown.

**Supplemental File 1**: Cuffdiff output for the deregulated transcripts.
