# Supplementary material for: A novel role for the 3′-5′ exoribonuclease Dis3L2 in controlling cell proliferation and tissue growth
Source: RNA Biol. 2016 Sep 14;13(12):1286–99. doi: 10.1080/15476286.2016.1232238 (PMC5207379; doi:10.1080/15476286.2016.1232238)
Supplement: Supplementary_Data.zip [file krnb-13-12-1232238-s001.zip › 5. Supplemental Figure 4.docx]

|  | Replicate | Paired reads  (millions) | Unpaired reads  (millions) | Total reads  (millions) | Reads mapped |
| --- | --- | --- | --- | --- | --- |
| *UAS-dis3L2^RNAi^* | 1 | 14.12 | 0.44 | 14.57 | 88.6% |
|  | 2 | 12.55 | 0.32 | 12.87 | 87.0% |
| *69B-Gal4* | 1 | 11.67 | 0.36 | 12.04 | 85.3% |
|  | 2 | 14.70 | 0.60 | 15.31 | 84.6% |
| Knockdown | 1 | 10.63 | 0.18 | 10.82 | 90.5% |
|  | 2 | 14.89 | 0.48 | 15.37 | 85.5% |

Summary of read counts and alignments for each RNA-seq replicate. Reads were aligned to chromosomes X, Y, 2, 3 and 4 of the FlyBase *Drosophila melanogaster* genome (r6.01) using TopHat v2.0.12 and Bowtie v2.2.3.

| Program | Non-default parameters | Effect |
| --- | --- | --- |
| TopHat2 | -i 20 | Minimum intron size (default 70bp). |
|  | -I 150000 | Maximum intron size (default 500000bp). |
|  | -r 100 | Expected inner distance between mate pairs (default 50). |
| Cufflinks | -u | More accurate weighting of reads mapping to multiple locations. |
|  | -N | Normalisation to upper quartile of number of fragments mapping to a locus instead of total number of fragments. Improves robustness of differential expression for lowly expressed transcripts. |
|  | -b | Includes a correction for fragment bias. |
|  | -compatible-hits-norm | Only fragments compatible with reference transcript counted in FPKM calculation. |
| Cuffmerge | None |  |
| Cuffquant | -u | More accurate weighting of reads mapping to multiple locations. |
|  | -b | Includes a correction for fragment bias. |
| Cuffdiff | -u | More accurate weighting of reads mapping to multiple locations. |
|  | -b | Includes a correction for fragment bias. |

Non-default parameters used for RNA-seq alignment and quantification.
