# Supplementary figures and images for: A novel role for the 3′-5′ exoribonuclease Dis3L2 in controlling cell proliferation and tissue growth
Source: RNA Biol. 2016 Sep 14;13(12):1286–99. doi: 10.1080/15476286.2016.1232238 (PMC5207379; doi:10.1080/15476286.2016.1232238)

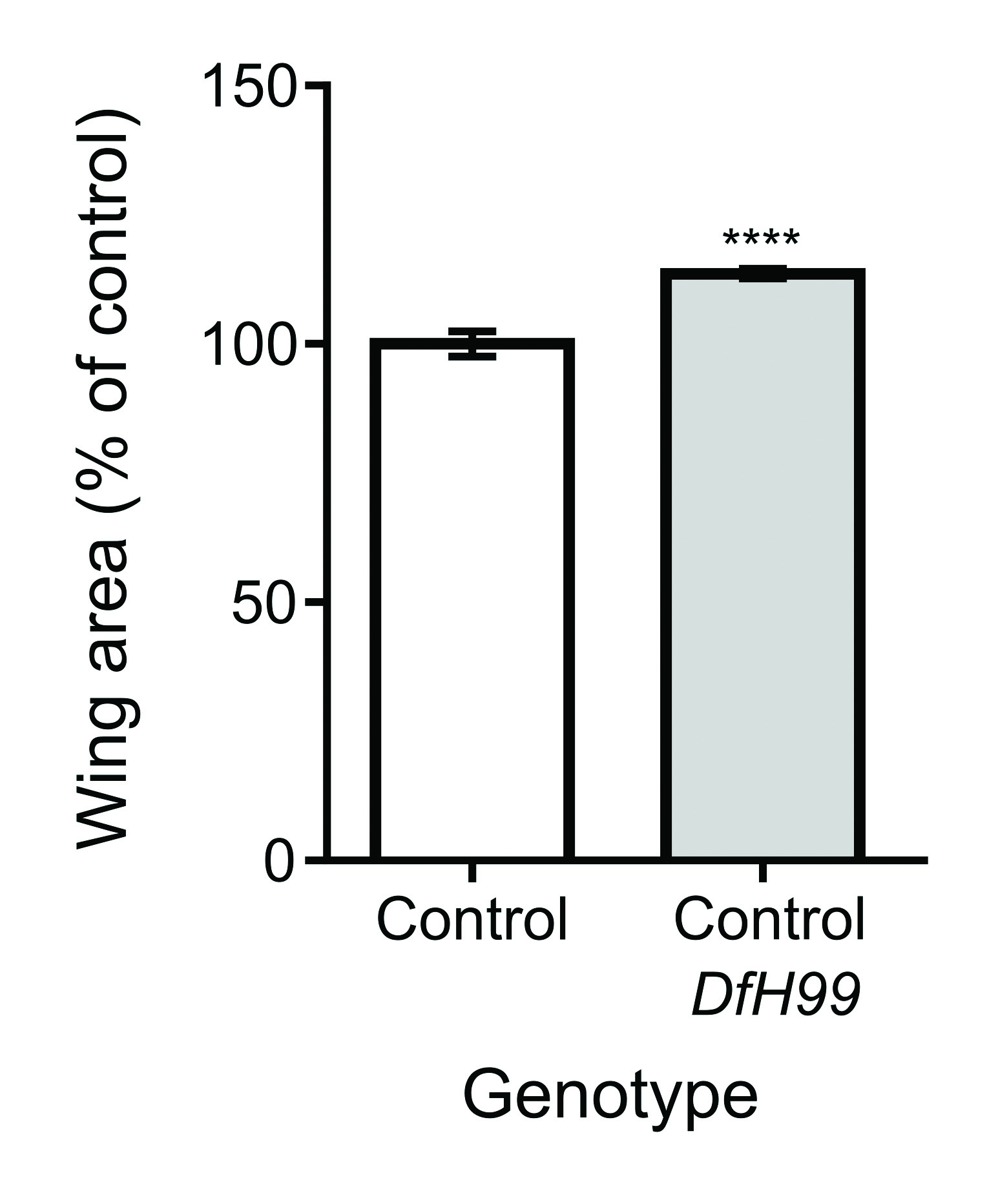

Supplement: Supplementary_Data.zip [file krnb-13-12-1232238-s001.zip › 10. Supplemental Figure 9.jpg]

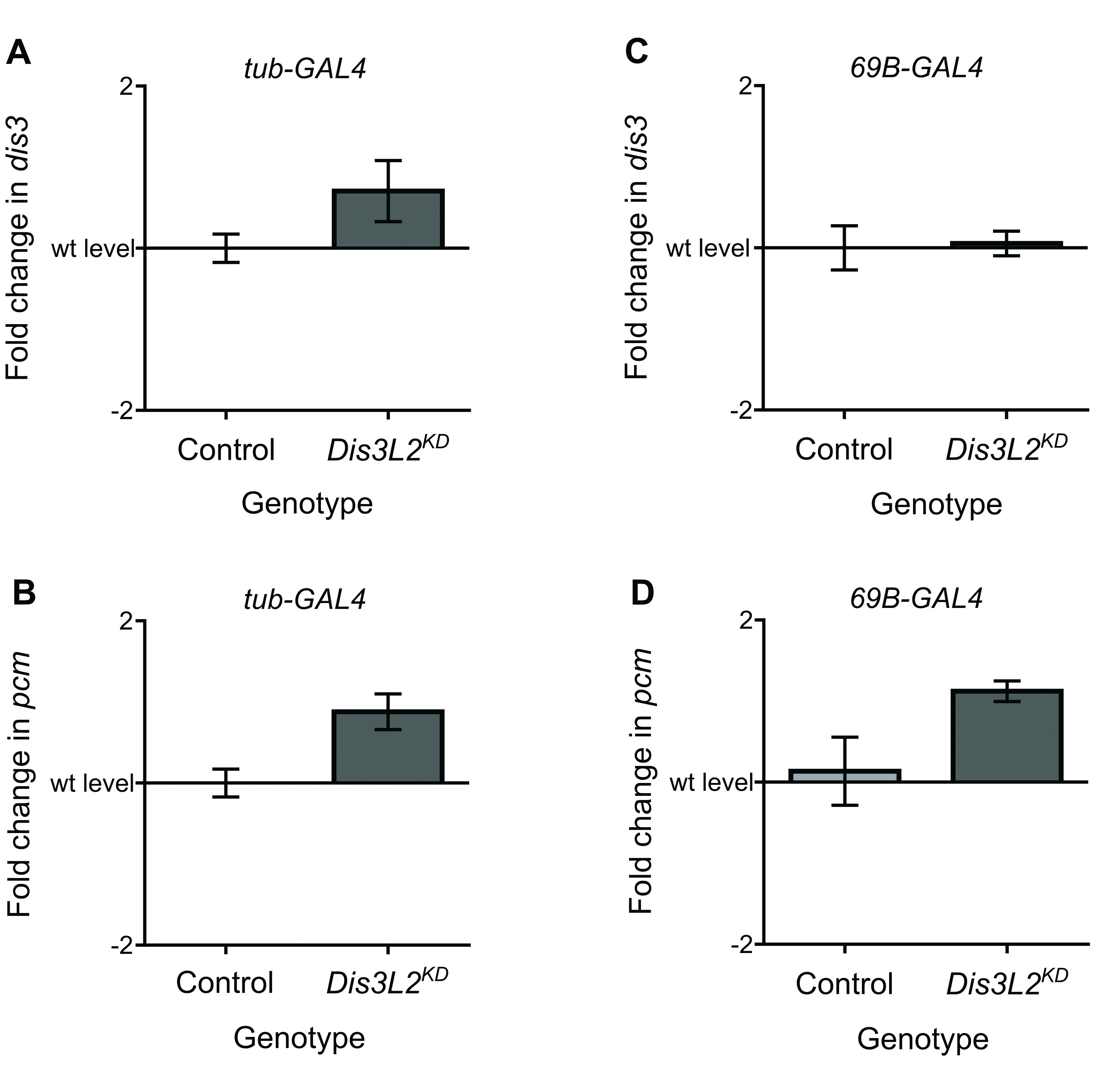

Supplement: Supplementary_Data.zip [file krnb-13-12-1232238-s001.zip › 11. Supplemental Figure 10.jpg]

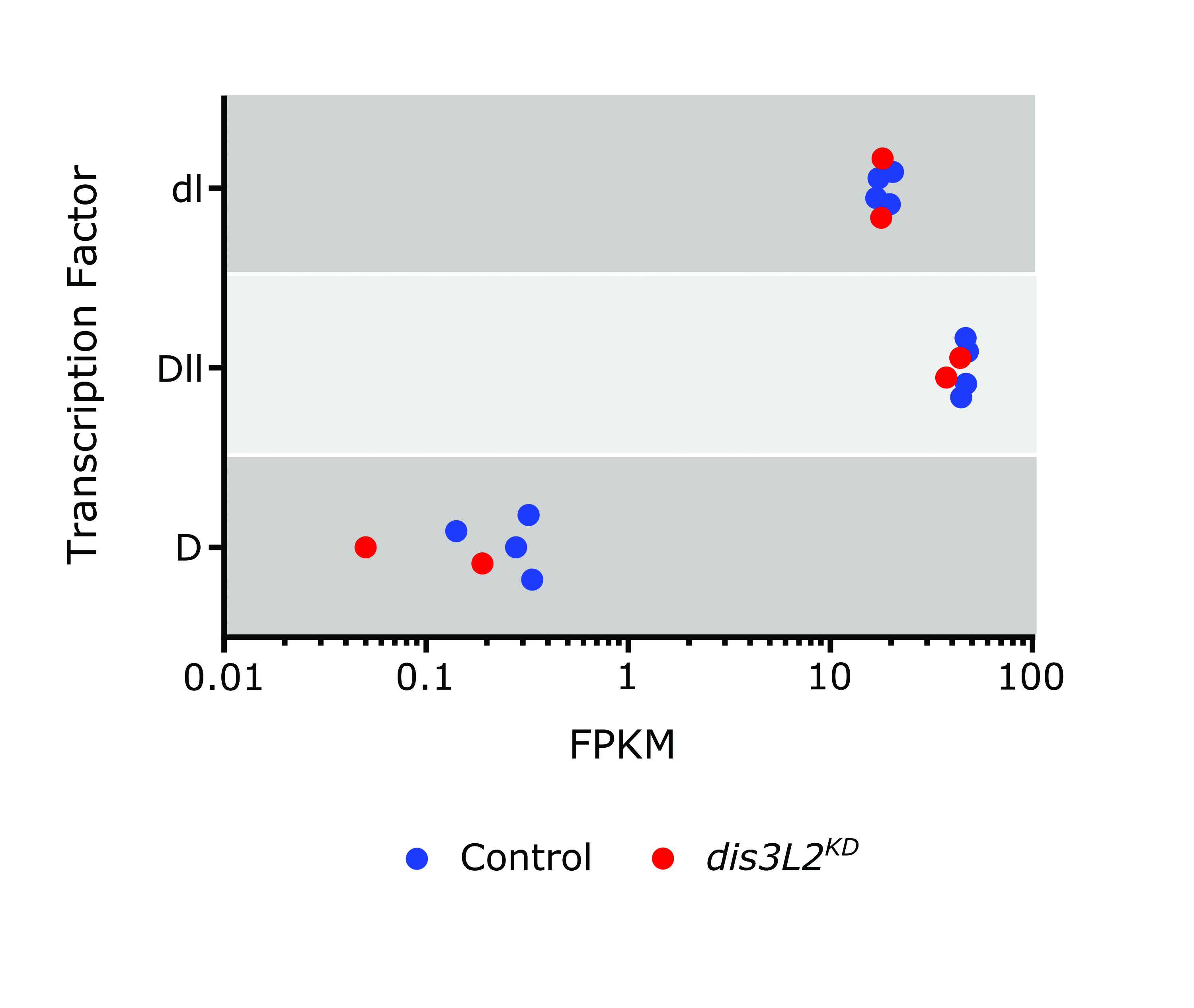

Supplement: Supplementary_Data.zip [file krnb-13-12-1232238-s001.zip › 12. Supplemental Figure 11.jpg]

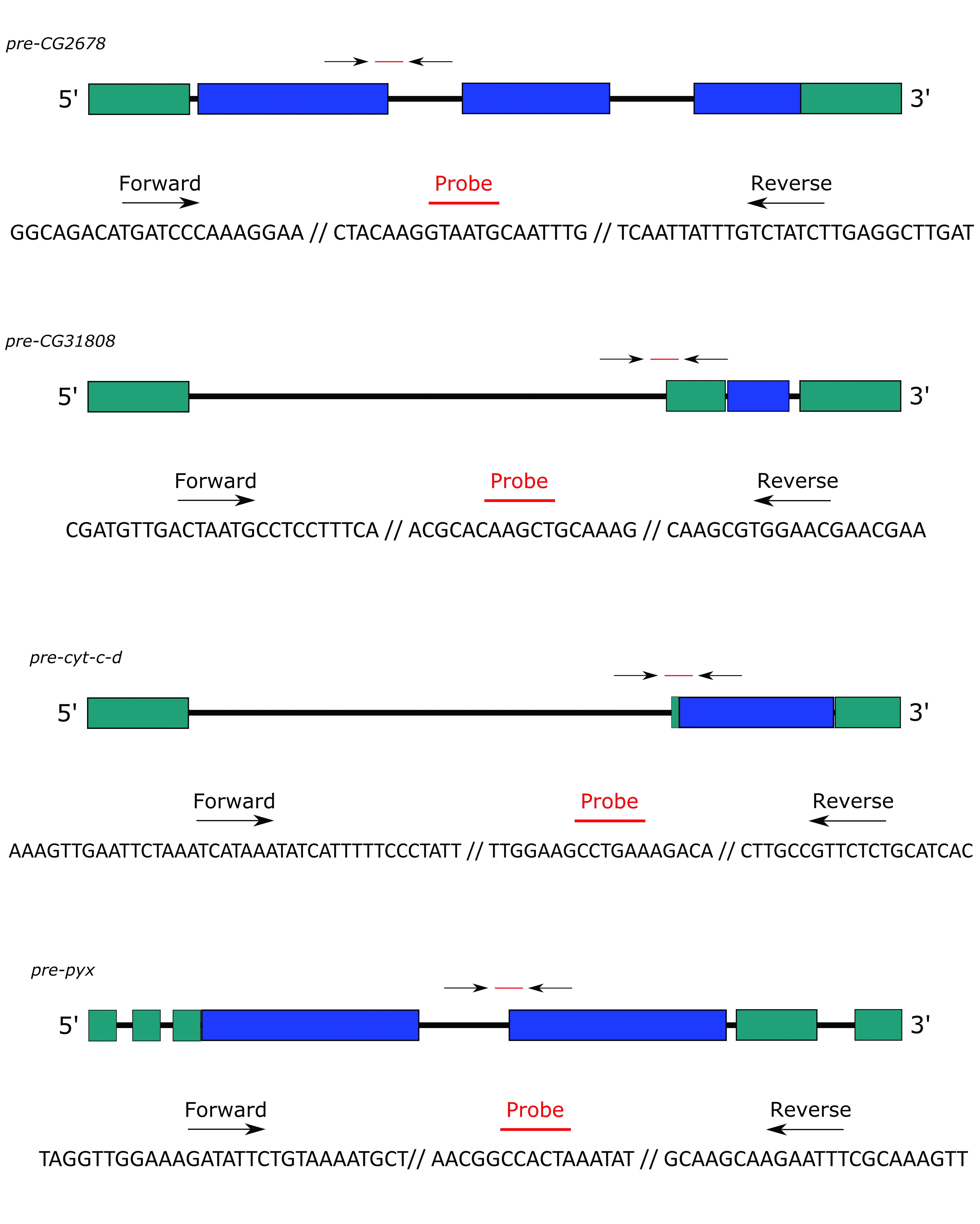

Supplement: Supplementary_Data.zip [file krnb-13-12-1232238-s001.zip › 13. Supplemental Figure 12.jpg]

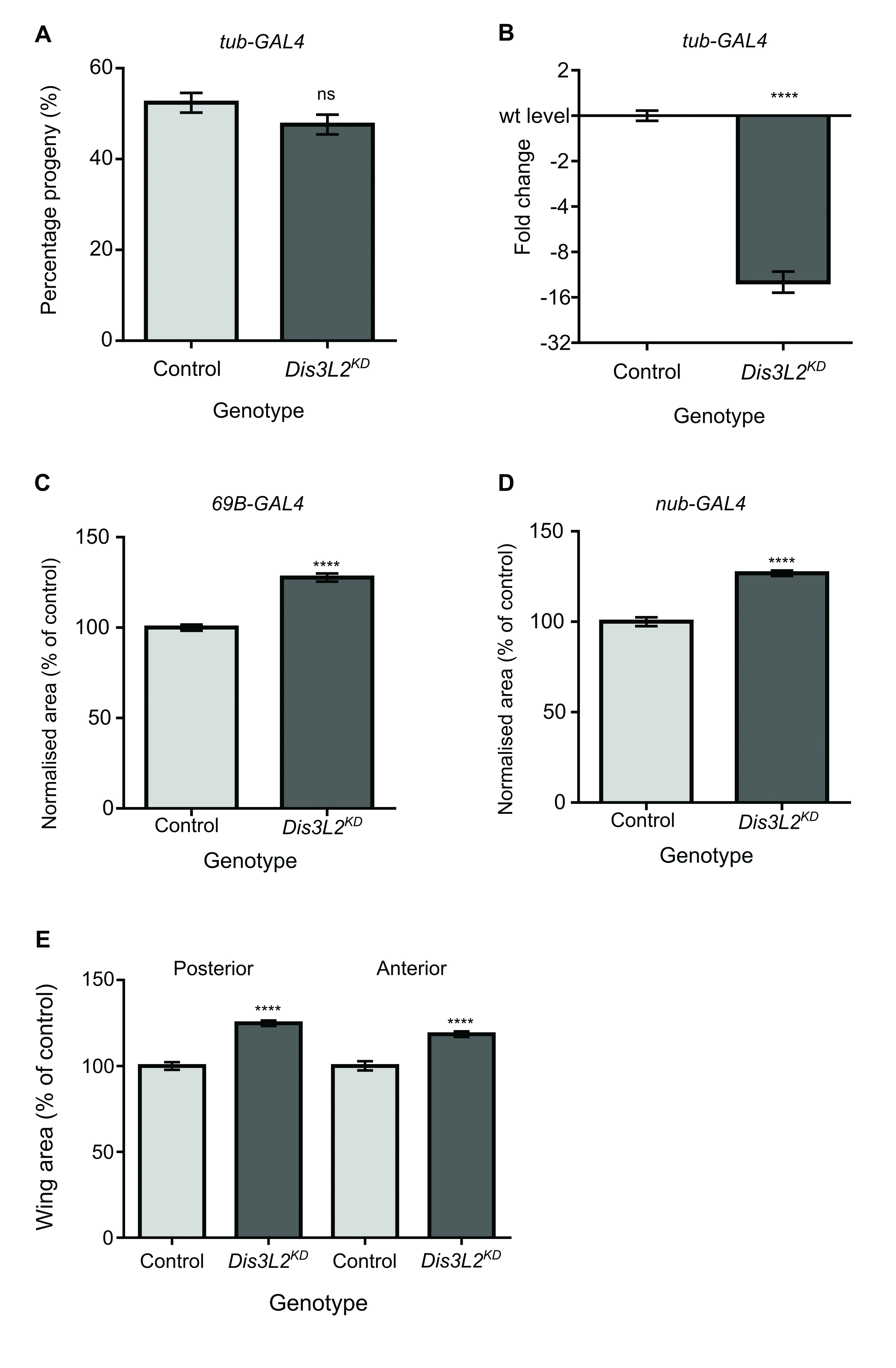

Supplement: Supplementary_Data.zip [file krnb-13-12-1232238-s001.zip › 2. Supplemental Figure 1.jpg]

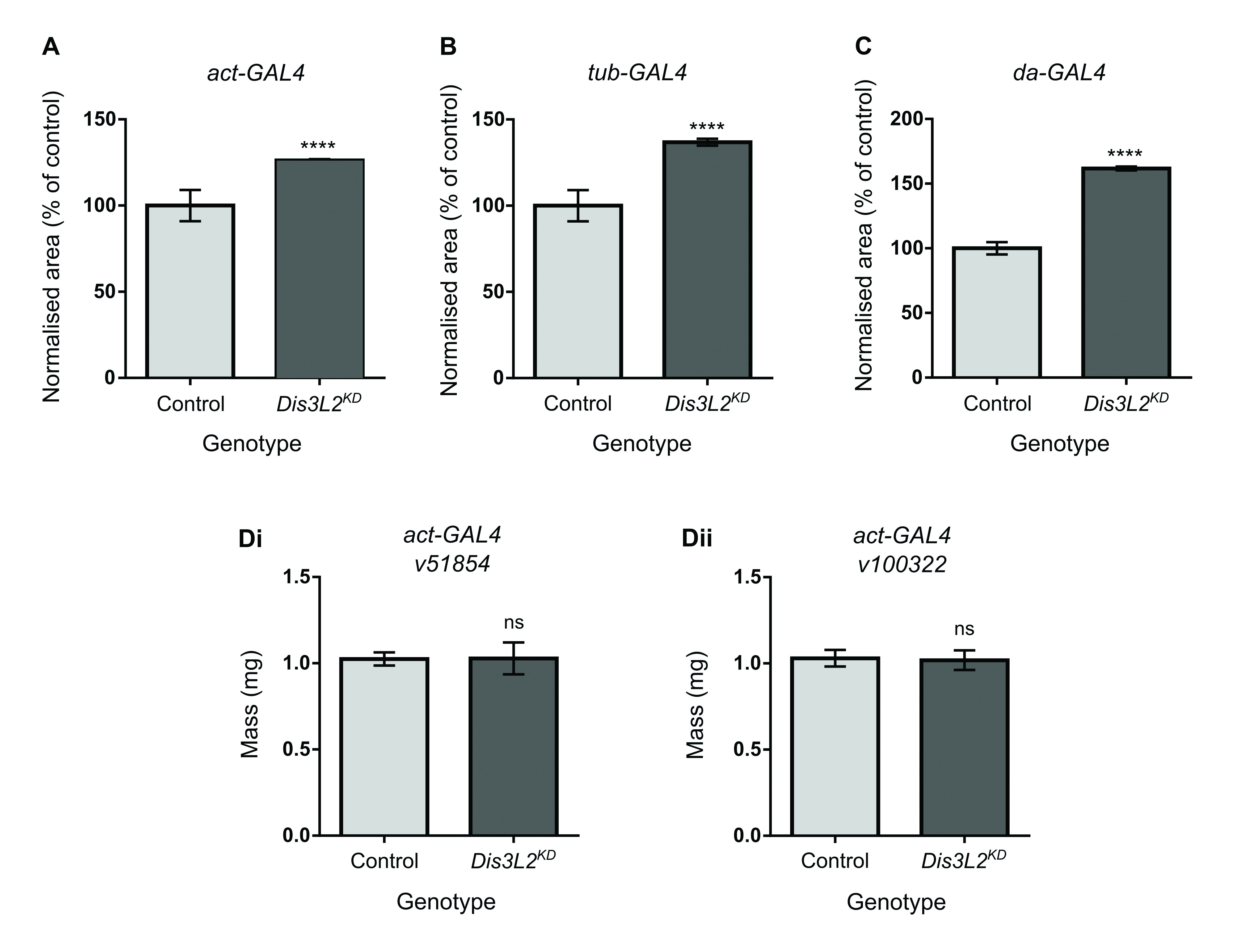

Supplement: Supplementary_Data.zip [file krnb-13-12-1232238-s001.zip › 3. Supplemental Figure 2.jpg]

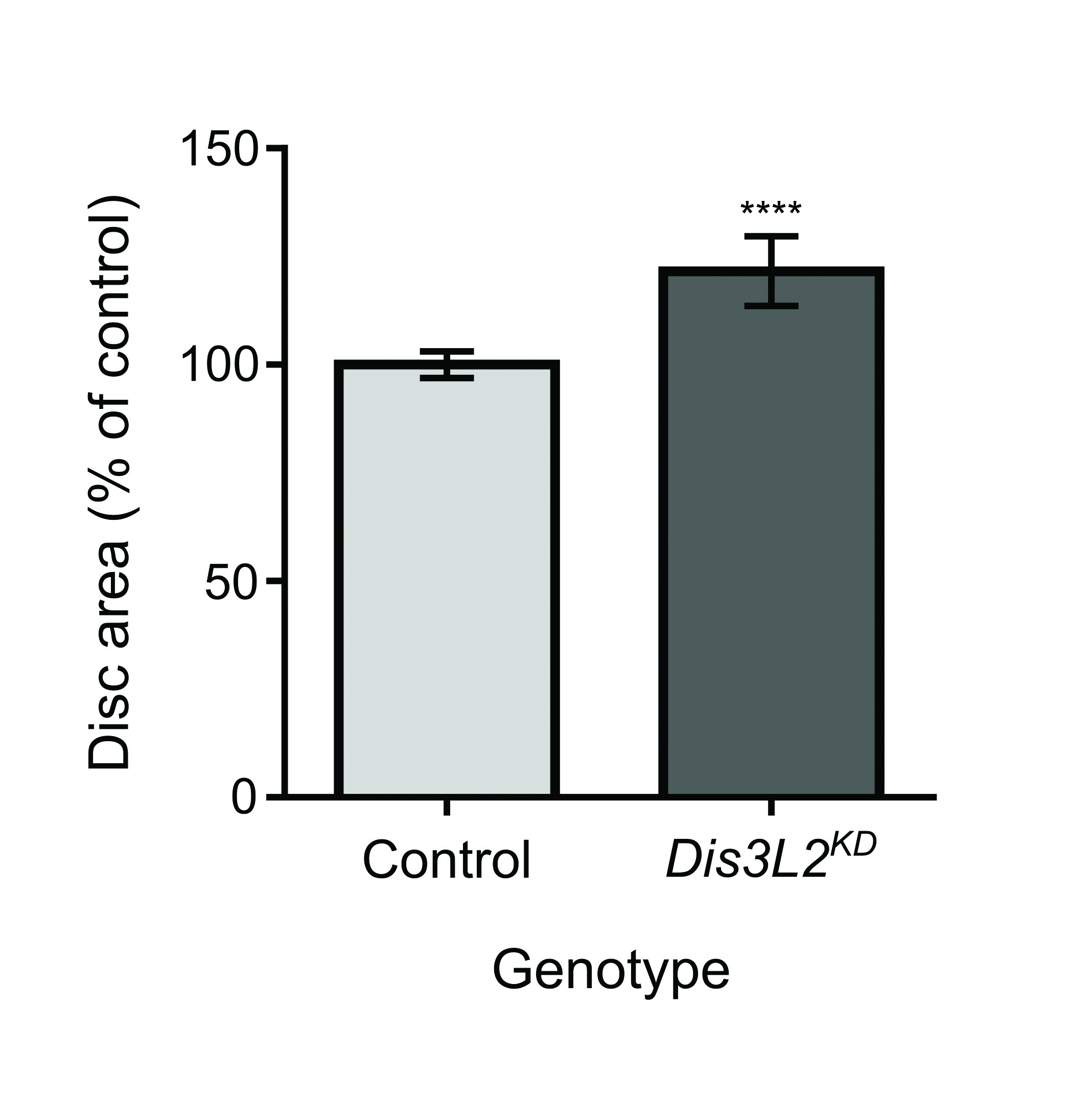

Supplement: Supplementary_Data.zip [file krnb-13-12-1232238-s001.zip › 4. Supplemental Figure 3.jpg]

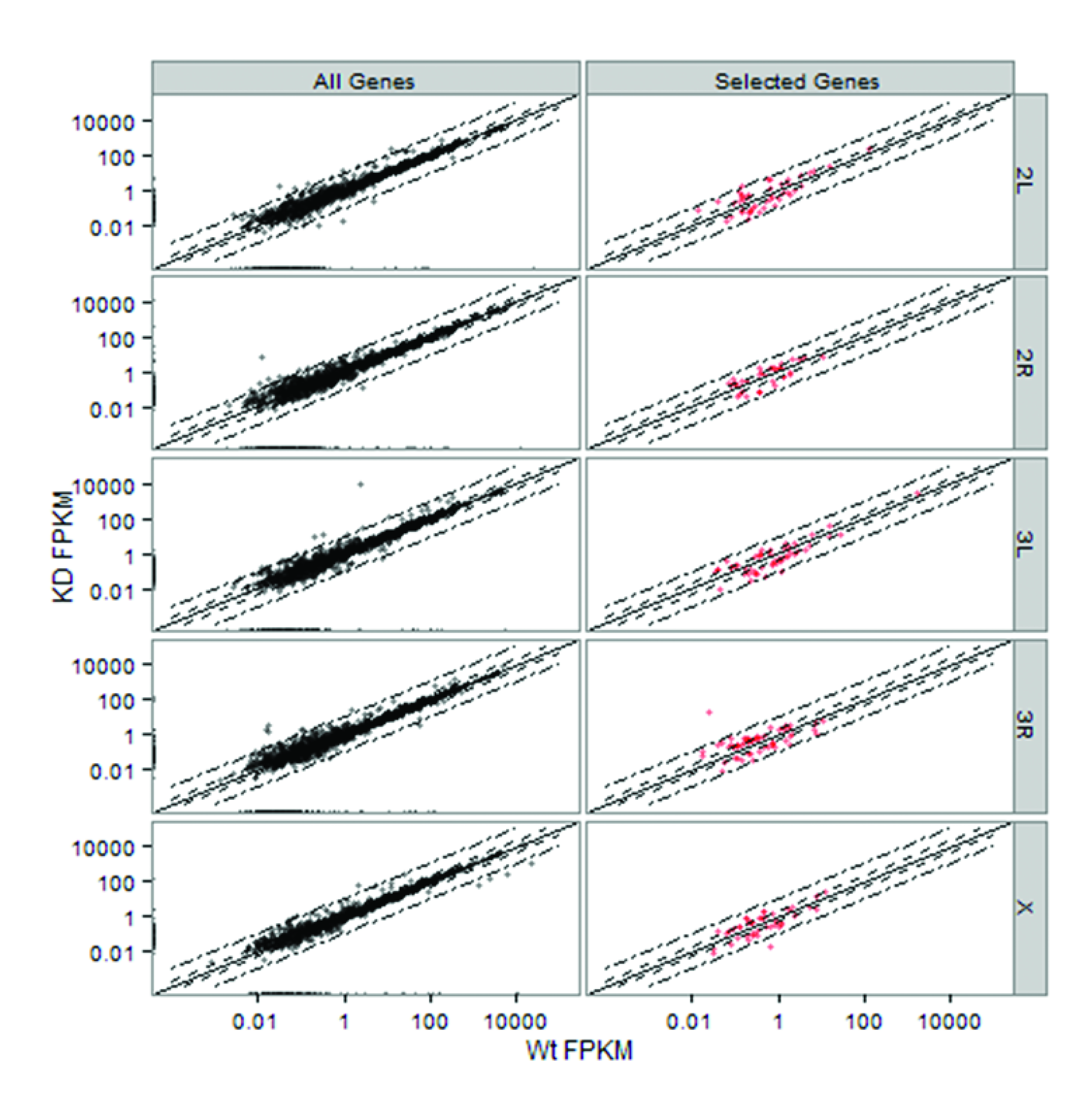

Supplement: Supplementary_Data.zip [file krnb-13-12-1232238-s001.zip › 6. Supplemental Figure 5.jpg]

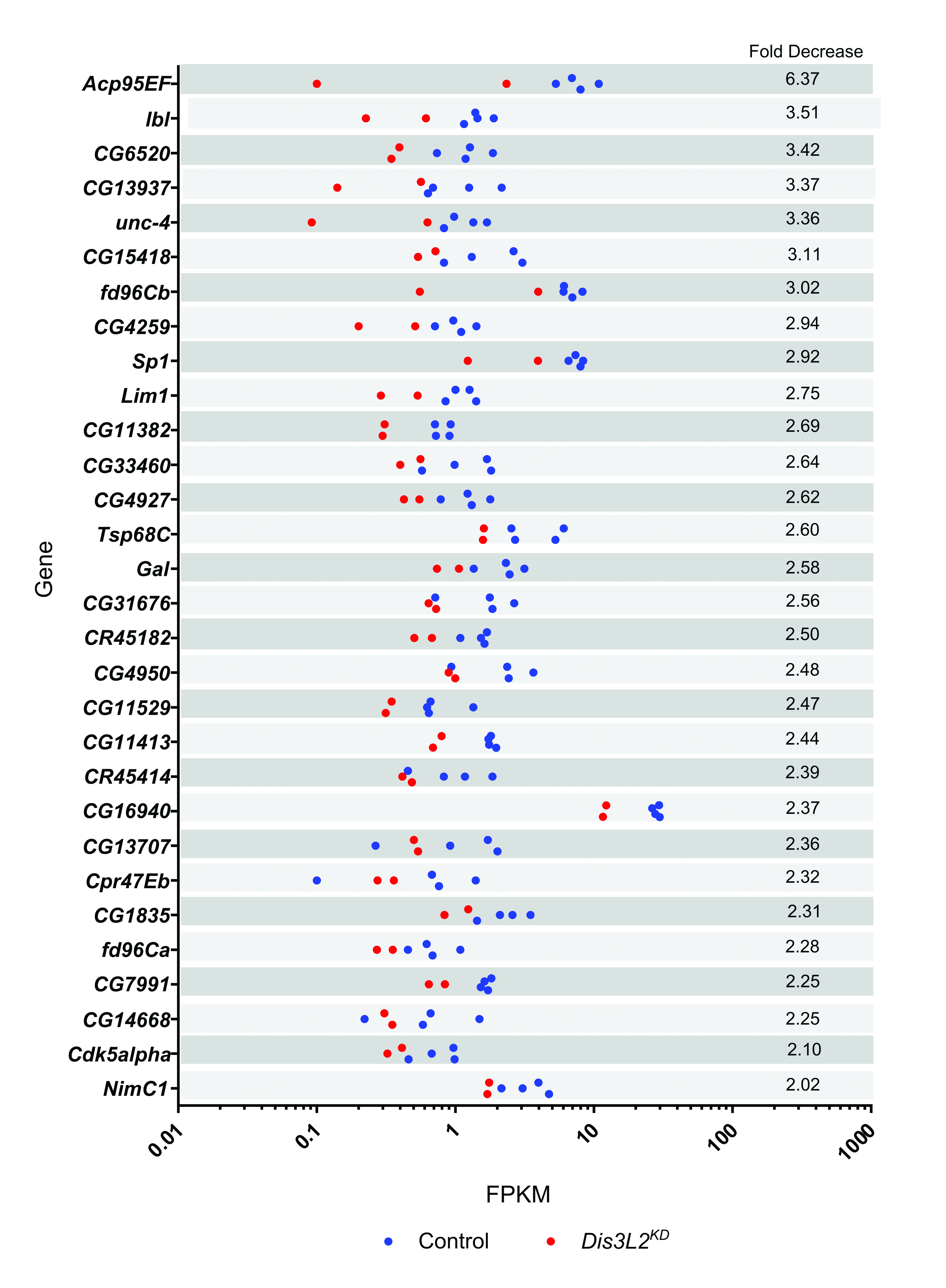

Supplement: Supplementary_Data.zip [file krnb-13-12-1232238-s001.zip › 7. Supplemental Figure 6.jpg]

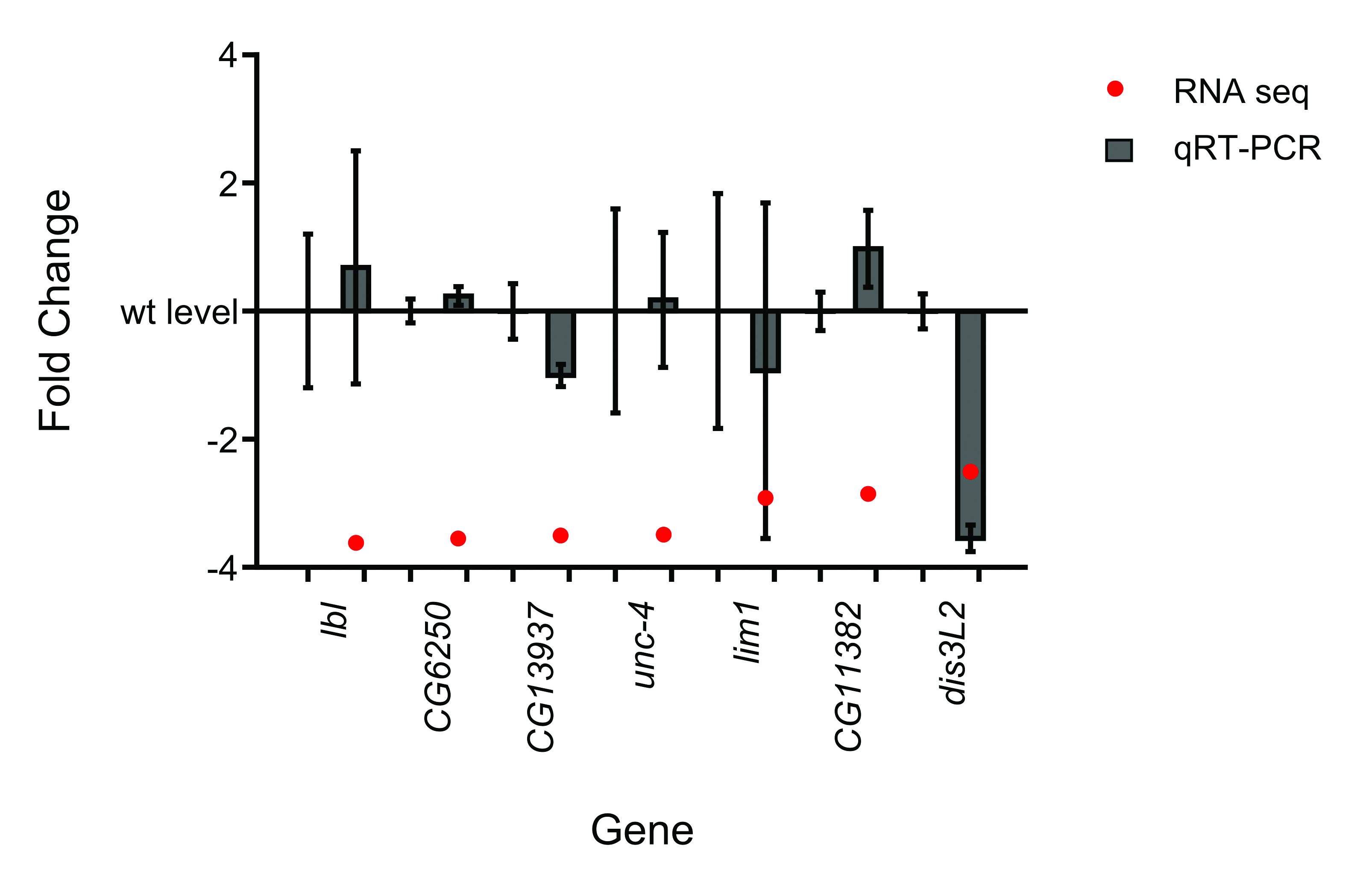

Supplement: Supplementary_Data.zip [file krnb-13-12-1232238-s001.zip › 8. Supplemental Figure 7.jpg]

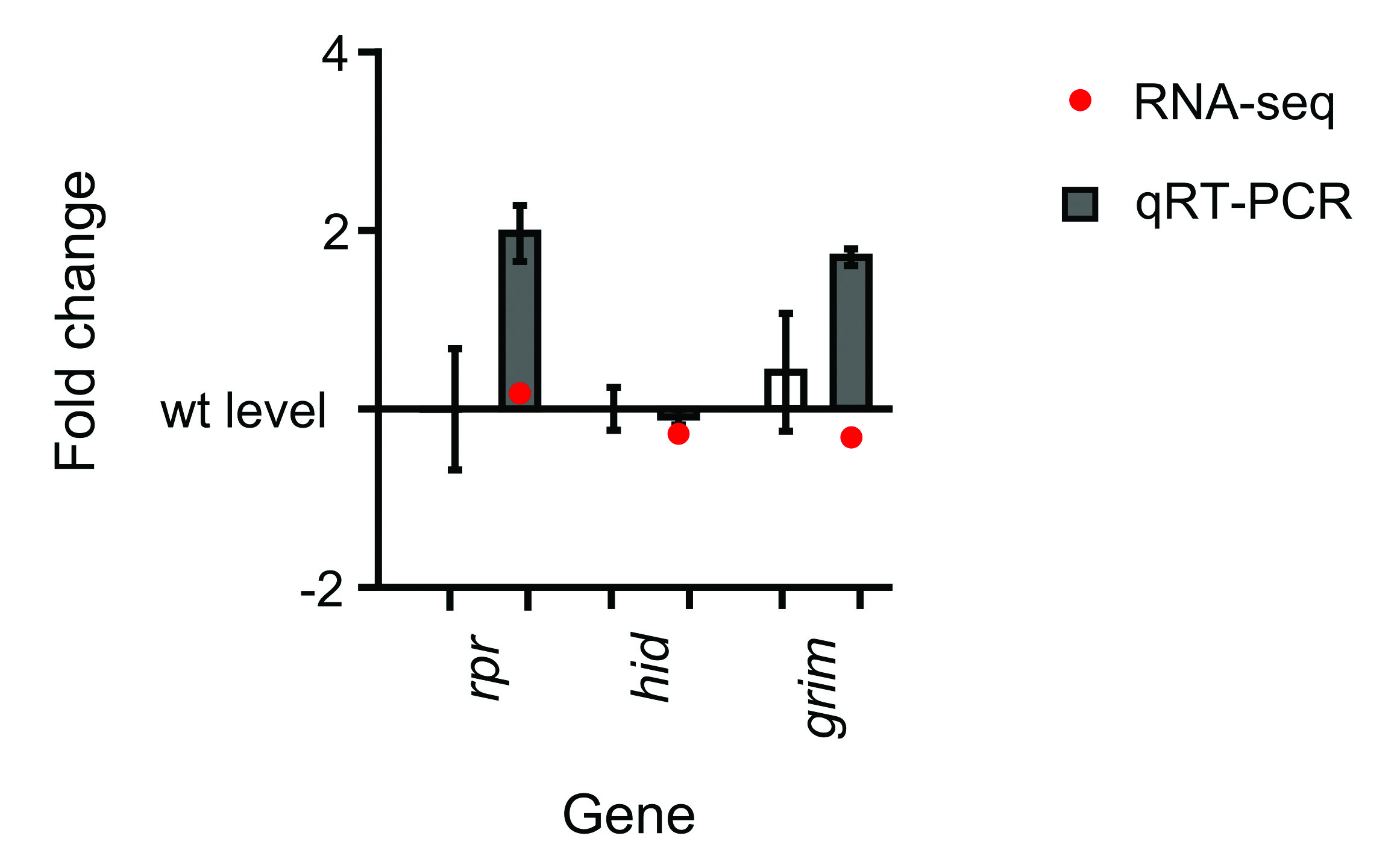

Supplement: Supplementary_Data.zip [file krnb-13-12-1232238-s001.zip › 9. Supplemental Figure 8.jpg]
